# Supplementary material for: Structure of a Rhs effector clade domain provides mechanistic insights into type VI secretion system toxin delivery
Source: Nat Commun. 2024 Oct 8;15:8709. doi: 10.1038/s41467-024-52950-x (PMC11461821; doi:10.1038/s41467-024-52950-x)
Supplement: Supplementary file 1 — Supplementary Information [file 41467_2024_52950_MOESM1_ESM.pdf]

Structure of a Rhs effector clade domain provides mechanistic insights into type VI secretion system toxin delivery

Brooke K. Hayes[1,2*], Marina Harper[1,2*], Hariprasad Venugopal[3], Jessica M. Lewis[1,2], Amy Wright[1,2], Han-Chung Lee[4], Joel R. Steele[4], David L. Steer[4], Ralf B. Schittenhelm[4], John D. Boyce[1,2#] and Sheena McGowan[1,2#]

Supplementary Information

Item	Description	Pg
Fig S1	Domain boundaries of Tse15.	2
Fig S2	Workflow for solving Tse15 wild-type structure and map to model fit.	3
Fig S3	Top AlphaFold2 model of Tse15.	4
Table S1	Metrics of Tse15 structures.	5
Fig S4	Tse15 clade domain is common to many bacterial Rhs effectors.	6
Table S2	List of homologues to the Tse15 N-terminal domain as determined by FoldSeek.	7
Fig S5	Access to the interior of the Rhs cage and interactions with the clade domain.	9
Table S3	Interactions between the clade and Rhs domains of Tse15.	10
Fig S6	Toxin difference maps of Tse15 and $Tse15_{NN}$ with fitted toxin residues.	11
Fig S7	Density of the toxin cleavage site of Tse15 and $Tse15_{NN}$.	12
Fig S8	Crosslinking mass spectrometry of Tse15.	13
Fig S9	$Tse15_{NN}$ construct design and purification.	14
Fig S10	Workflow for solving $Tse15_{NN}$ structure and map to model fit.	15
Fig S11	Identification and mutation of the clade autocleavage motif.	16
Table S4	Conservation of the clade autocleavage motif in across N-terminal domain homologues.	17
Fig S12	Pulldown of native VgrG15 with recombinant Tse15.	18
Fig S13	The VgrG15 N-terminal domain is highly conserved as compared to the N-terminal domains of the other *A. baumannii* AB307 VgrG proteins.	19
Fig S14	Example of one two-hybrid experiment showing selected alanine substitutions.	20
Fig S15	Tsi15 is not required to protect the host cell.	21
Fig S16	Peptide signatures of Rhs effectors and their cognate VgrG within the secretome of T6SS active *A. baumannii*.	22
Table S5	MS/MS proteomic data from secretome analysis including whole cell lysate and supernatant samples from *A. baumannii* AB307-0294.	23
Table S6	Bacterial strains and plasmids used in this study.	24
Table S7	Primers used in this study.	27

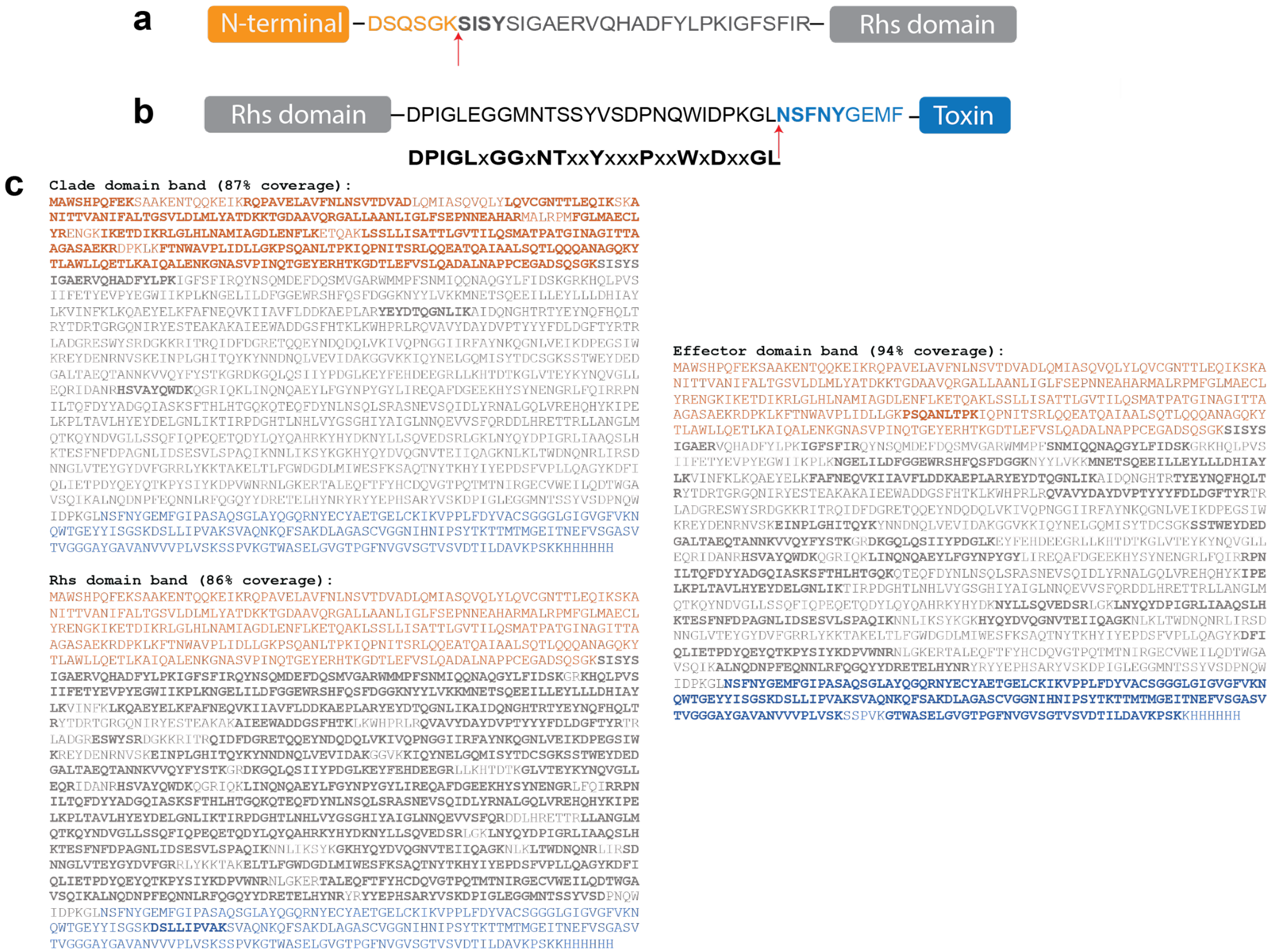

Supp Figure 1. Domain boundaries of Tse15. a) N-terminal sequencing of the Rhs domain: N-terminal domain is orange, Rhs domain is grey. Bold residues indicate residues identified by N-terminal sequencing. **b)** N-terminal sequencing of the toxin domain: Rhs domain is grey and toxin domain is blue. Blue bold residues indicate residues identified by N-terminal sequencing. Residues below show alignment of consensus sequence. **c)** Mass spectrometry peptide fingerprinting of excised bands for each domain from SDS-PAGE. Identity of the excised band is indicated at the top with percentage peptide coverage for each domain. Domains are coloured as follows: N-terminal clade residues are orange, Rhs domain residues grey and toxin domain residues blue. Residues shown in bold indicate peptides identified.

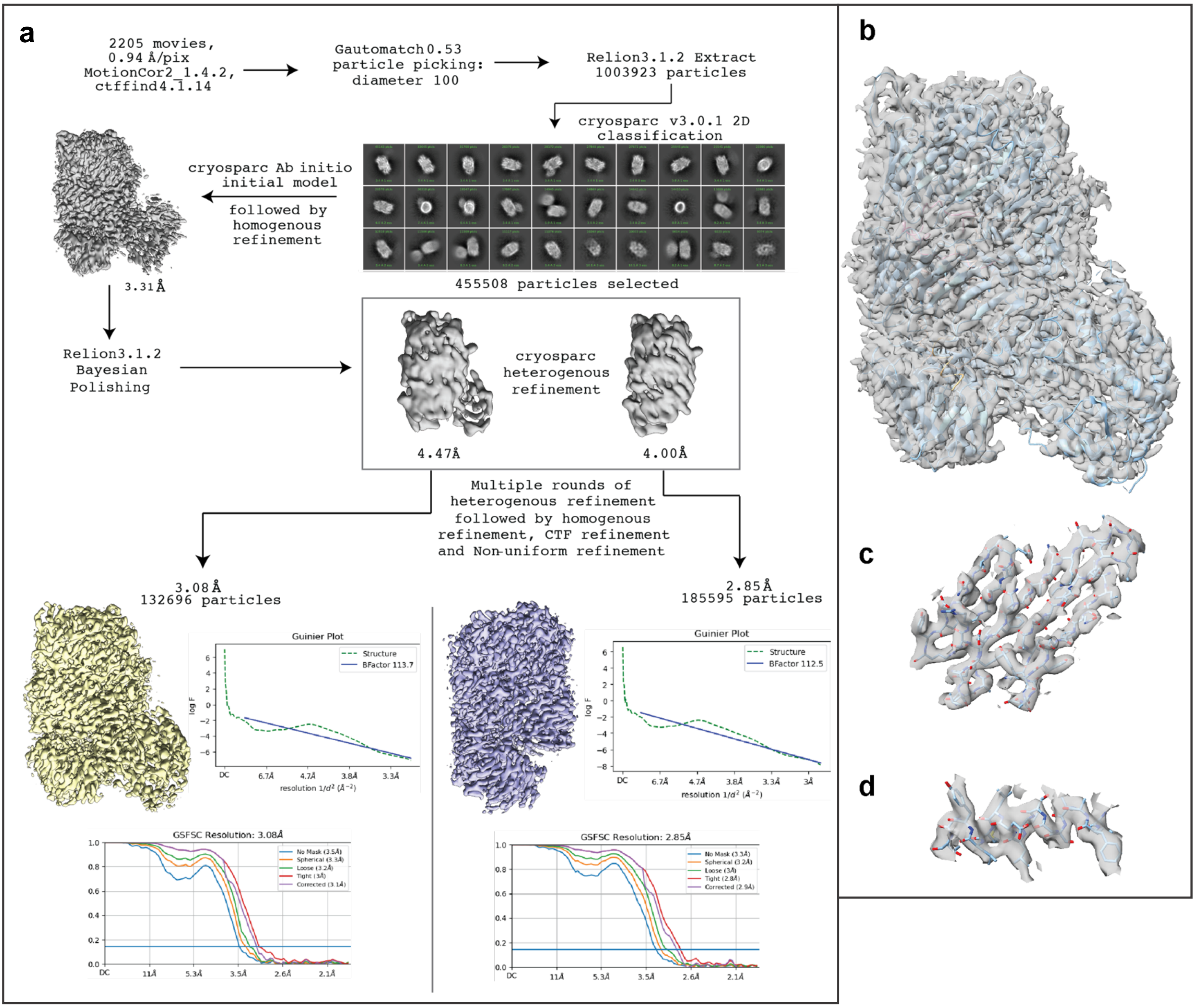

Supp Figure 2. Workflow for solving Tse15 wild-type structure and map to model fit. (Left box, a). Particles were picked using Gautomatch, then extracted using Relion. Cryosparc was used for 2D classification, an *ab initio* model and homogenous refinement that produced a 3.31 Å map. Particles were polished in Relion, and then used for heterogenous refinement in Cryosparc to produce two particle classes at 4.47 and 4 Å. These were separately refined further using heterogenous refinement, CTF refinement and non-uniform refinement to produce a Tse15 map with the clade domain at 3.08 Å and a Tse15 map without the clade domain at 2.85 Å. **Right box** shows map to model fit **(b)** Tse15 fit to model shown as cartoon (contour 0.671); (**c**) Rhs domain β-sheet residues 900 – 952 (contour 1.2) and (**d**) clade domain α-helix residues 67 – 82 (contour 0.8). Both **c)** and **d)** are shown as sticks and coloured by atom.

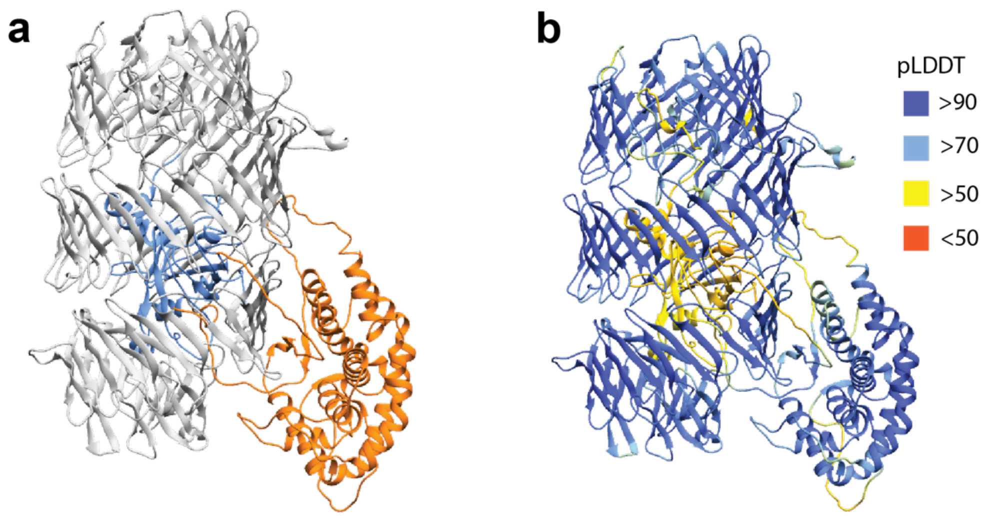

Supp Figure 3. Top AlphaFold2 model of Tse15. a) model with the different domains coloured: orange is N-terminal domain, grey Rhs domain and blue toxin domain. **b)** pLDDT score mapped onto the Tse15 model.

Supp Table 1. Cryo-EM data collection, refinement and validation statistics.

	**Tse15** (EMDB-42792) (PDB 8UY4)	**$Tse15_{NN}$** (EMDB-42775) (PDB 8UXT)
**Data collection and processing**		
Magnification (kx)	150	130
Voltage (kV)	200	300
Electron exposure (e–/Å^2)	50	60
Defocus range (μm)	0.5 to 1.2	0.4 to 1.2
Pixel size (Å)	0.94	0.65
Symmetry imposed	C1	C1
Initial particle images (no.)	1003923	4549557
Final particle images (no.)	132696	1163105
Map resolution (Å)<br>FSC threshold	3.08<br>0.143	1.89<br>0.143
Map resolution range (Å)	3.08 – 10.96	1.77 – 7.05
**Refinement**		
Initial model used (PDB code)	-	-
Model resolution (Å)<br>FSC threshold	3.08<br>0.143	1.89<br>0.143
Model resolution range (Å)	-	-
Map sharpening *B* factor (Å^2)	-	-
Model composition<br>Non-hydrogen atoms<br>Protein residues<br>Ligands	 11464<br>1454<br>0	 11270<br>1414<br>0
B factors (Å^2)<br>Protein<br>Ligand	 134.61<br>-	 47.28<br>-
R.m.s. deviations<br>Bond lengths (Å)<br>Bond angles (°)	 0.03<br>0.756	 0.003<br>0.480
Validation<br>MolProbity score<br>Clashscore<br>Poor rotamers (%)	 3.86<br>7.71<br>1.55	 1.72<br>4.58<br>1.45
Ramachandran plot<br>Favored (%)<br>Allowed (%)<br>Disallowed (%)	 92.97<br>6.61<br>1.55	 95.01<br>4.49<br>1.45

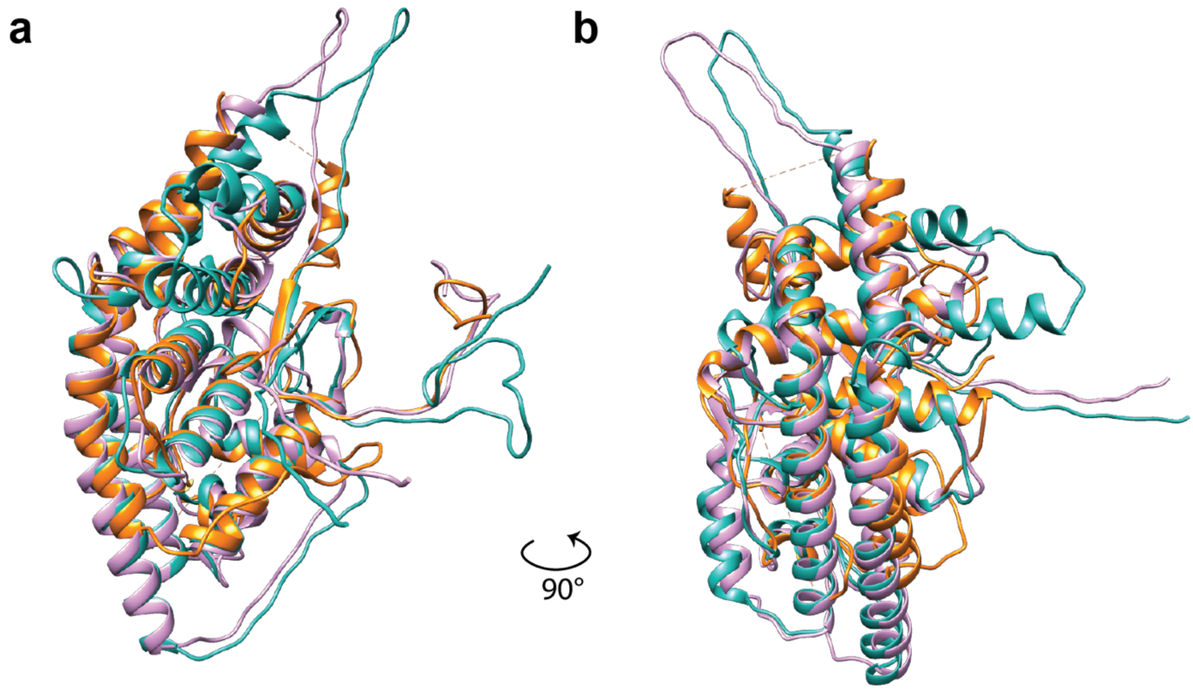

Supp Figure 4. Tse15 clade domain is common to many bacterial Rhs effectors. a) Cartoon alignment of the Tse15 N-terminal domain (orange) with AlphaFold2 structures of the Rhs N-terminal domains from *Pseudomonas sp.* WS 5354 (pink, RMSD 7.2 Å: NCBI, WP_169966139.1) and *Burkholderia sp.* AU6039 (teal, RMSD 9.4 Å, NCBI: NKFK01000008.1). **b)** depicts **a)** rotated 90 degrees.

Supp Table 2. List of homologues to the Tse15 N-terminal domain as determined by FoldSeek[1].

Target	Description	Scientific Name	Prob.	Seq. Id. (%)	E-Value	Position in query
AF-A0A6I4HT81-F1-model_v4	RHS repeat protein	*Acinetobacter baumannii*	1	100.0	9.70E-46	1-333
AF-L9LR64-F1-model_v4	YD repeat protein (3 repeats)	*Acinetobacter sp.* OIFC021	1	94.5	1.61E-44	1-333
AF-A0A7Z2HG68-F1-model_v4	Rhs family protein	*Acinetobacter baumannii*	1	98.1	9.45E-45	1-333
AF-N9DJH2-F1-model_v4	Uncharacterized protein	*Acinetobacter bereziniae* LMG 1003 = CIP 70.12	1	60.1	3.95E-34	1-326
AF-A0A1H7XNA3-F1-model_v4	YD repeat-containing protein	*Acinetobacter sp.* DSM 11652	1	58.7	2.48E-33	1-331
AF-A0A429MRQ6-F1-model_v4	RHS repeat protein	*Acinetobacter baumannii*	1	100.0	6.89E-24	85-333
AF-A0A7Y1FLN0-F1-model_v4	RHS repeat protein	*Pseudomonas sp.* WS 5078	1	23.9	1.30E-14	1-332
AF-A0A3M4WEW4-F1-model_v4	RHS repeat containing protein	*Pseudomonas cichorii*	1	25.9	3.42E-14	9-325
AF-A0A519DSR3-F1-model_v4	RHS repeat protein	*Pseudomonas sp.*	1	25.5	4.80E-14	8-325
**AF-A0A7Y1G8B5-F1-model_v4**	**RHS repeat protein**	***Pseudomonas sp.* WS 5354**	**1**	**24.7**	**6.41E-14**	**2-325**
AF-A0A1I0LVF7-F1-model_v4	YD repeat-containing protein	*Burkholderia cepacia*	1	24.3	4.80E-14	1-311
AF-A0A4R7US80-F1-model_v4	Uncharacterized protein	*Pseudomonas helmanticensis*	1	25.3	3.59E-14	5-325
AF-A0A7C1WQ62-F1-model_v4	RHS repeat protein	*Pseudomonas graminis*	1	22.8	3.49E-13	1-333
AF-A0A3P0RKZ5-F1-model_v4	RHS repeat protein	*Burkholderia sp.* Bp9004	1	23.4	3.17E-13	1-311
AF-A0A519FHV8-F1-model_v4	RHS repeat protein	*Pseudomonas sp.*	1	23.7	1.73E-12	12-322
AF-A0A107A4U9-F1-model_v4	Uncharacterized protein	*Burkholderia stagnalis*	1	22.8	1.35E-12	7-325
AF-J2TDQ0-F1-model_v4	Rhs family protein	*Variovorax sp.* CF313	1	23.6	3.75E-12	1-327
AF-A0A365QG90-F1-model_v4	RHS family protein	*Burkholderia reimsis*	1	21.5	3.75E-12	15-333
AF-A0A7X2BG59-F1-model_v4	RHS repeat protein	*Pseudomonas sp.* FSL R10-0765	1	22.7	9.40E-12	12-332
AF-A0A837MJY1-F1-model_v4	Uncharacterized protein	*Pseudomonas helleri*	1	24.0	6.85E-11	12-324
AF-A0A6I4HSU2-F1-model_v4	Type IV secretion protein Rhs	*Acinetobacter baumannii*	1	22.5	2.04E-11	1-322
AF-A0A7Y1HMJ9-F1-model_v4	RHS repeat protein	*Pseudomonas lundensis*	1	23.8	5.12E-11	21-326
AF-A0A1H7LLH1-F1-model_v4	Uncharacterized protein	*Variovorax sp.* YR750	1	24.9	2.36E-11	8-333
AF-A0A7G2MP46-F1-model_v4	Uncharacterized protein	*Pseudomonas sp.*	1	23.3	2.19E-10	12-324
AF-A0A4R8LT33-F1-model_v4	YD repeat-containing protein	*Paraburkholderia rhizosphaerae*	1	22.1	1.08E-09	5-333
AF-A0A1H7ETW9-F1-model_v4	Uncharacterized protein	*Variovorax sp.* OK202	1	25.2	3.65E-11	1-333
AF-A0A6L5HR91-F1-model_v4	Uncharacterized protein	*Pseudomonas helleri*	1	23.3	2.30E-10	10-318
AF-A0A3P0RGT4-F1-model_v4	Uncharacterized protein	*Burkholderia sp.* Bp9004	1	23.5	3.91E-10	31-333
AF-A0A4Y8HF87-F1-model_v4	YD repeat-containing protein	*Pseudomonas sp.* URIL14HWK12:I1	1	20.8	2.59E-09	7-315
AF-A0A1W1Y1F1-F1-model_v4	YD repeat-containing protein	*Andreprevotia lacus* DSM 23236	1	22.8	2.59E-09	8-331
AF-A0A071MKM1-F1-model_v4	Sugar-binding protein	*Burkholderia cenocepacia*	1	19.9	1.41E-08	17-321
**AF-A0A228NXD1-F1-model_v4**	**Type IV secretion protein Rhs**	***Burkholderia sp.* AU6039**	**1**	**19.8**	**1.45E-09**	**7-333**

AF-N9MJ93-F1-model_v4	Uncharacterized protein	*Acinetobacter sp.* ANC 4105	1	83.4	3.34E-15	115-332
AF-A0A358GY28-F1-model_v4	Type IV secretion protein Rhs	*Acinetobacter sp.*	1	20.2	4.29E-08	17-327
AF-A0A3P0RK35-F1-model_v4	RHS repeat protein	*Burkholderia sp.* Bp9004	1	17.5	1.83E-07	17-311
AF-N8WS81-F1-model_v4	Uncharacterized protein	*Acinetobacter guillouiae* NIPH 991	1	18.9	2.34E-07	12-326
AF-A0A8B5S1J0-F1-model_v4	RHS repeat protein	*Acinetobacter bereziniae*	1	60.2	7.03E-12	106-333
AF-A0A4R6Y000-F1-model_v4	YD repeat-containing protein	*Hydromonas duriensis*	1	17.8	3.13E-07	1-324
AF-A0A009GBA8-F1-model_v4	RHS Repeat family protein	*Acinetobacter baumannii* 118362	1	14.9	3.79E-07	17-320
AF-A0A429MUJ9-F1-model_v4	RHS repeat protein	*Acinetobacter baumannii*	1	15.4	6.78E-07	17-320
AF-A0A4R3W625-F1-model_v4	YD repeat-containing protein	*Pseudomonas sp.* LP_8_YM	1	17	9.27E-06	16-315
AF-A0A3N7ZDS0-F1-model_v4	RHS repeat protein	*Burkholderia sp.* Bp9142	1	19.4	1.07E-05	7-328
AF-A0A7X1WP70-F1-model_v4	Rhs family protein	*Pseudomonas sp.* FSL R10-0399	1	18.3	2.28E-06	8-324
AF-A0A6P2ZV05-F1-model_v4	Sugar-binding protein	*Burkholderia contaminans*	1	17.8	3.11E-05	27-325
AF-L9MNM7-F1-model_v4	Uncharacterized protein	*Acinetobacter sp.* WC-743	1	61.7	1.97E-06	1-159
AF-A0A3S0B389-F1-model_v4	RHS repeat protein	*Variovorax sp.* 679	1	22.3	5.84E-05	94-302
AF-A0A3P0RI29-F1-model_v4	RHS repeat protein	*Burkholderia sp.* Bp9004	1	18.9	1.50E-05	7-299
AF-A0A1H7QHI4-F1-model_v4	Uncharacterized protein	*Variovorax sp.* YR750	1	23.9	7.09E-05	1-299
AF-A0A7L7VH69-F1-model_v4	Uncharacterized protein	*Pseudomonas putida*	1	16.1	8.79E-04	17-326
AF-A0A0D0MNU1-F1-model_v4	Uncharacterized protein	*Variovorax paradoxus*	1	20.4	7.60E-04	21-290
AF-A0A6N8T242-F1-model_v4	Sugar-binding protein	*Burkholderia sp.* 4701	1	13.9	1.47E-04	25-302
AF-J3CCY2-F1-model_v4	Rhs family protein	*Variovorax sp.* CF313	1	23	1.02E-03	99-333
AF-A0A7X1W4E1-F1-model_v4	Rhs family protein	*Pseudomonas sp.* FSL R10-0765	1	17.4	1.50E-03	26-333
AF-A0A2U9SMH5-F1-model_v4	Uncharacterized protein	*Burkholderia sp.* JP2-270	1	16.1	9.23E-04	21-326
AF-A0A4R0X6U5-F1-model_v4	Uncharacterized protein	*Paraburkholderia steynii*	1	16.5	1.43E-03	13-333
AF-A0A105TLI1-F1-model_v4	Uncharacterized protein	*Pseudomonas sp.* TAD18	0.93	14.2	8.33E-02	26-333
AF-A0A143IY85-F1-model_v4	Uncharacterized protein	*Acinetobacter pittii*	0.72	14.6	2.20E-01	21-333
AF-S9QIT6-F1-model_v4	Filamentous hemagglutinin	*Cystobacter fuscus* DSM 2262	0.6	11.5	6.37E-01	17-333
AF-A0A3N8B1N8-F1-model_v4	RHS family protein	*Burkholderia sp.* Bp9143	0.57	19.4	1.32E+00	152-332
AF-E0SGL7-F1-model_v4	Putative deoxyribonuclease RhsC	*Dickeya dadantii* 3937	0.54	11.4	5.78E-01	68-333
AF-A0A6I3T6V6-F1-model_v4	Type IV secretion protein Rhs	*Pseudoduganella buxea*	0.44	19	1.38E+00	146-333
AF-N9RKC7-F1-model_v4	Uncharacterized protein	*Acinetobacter sp.* NIPH 2100	0.35	8.6	1.52E+00	34-333
AF-A0A7H2WCG2-F1-model_v4	Ntox15 domain-containing protein	*Acinetobacter seifertii*	0.3	12.9	2.04E+00	26-333
AF-A0A378QIP5-F1-model_v4	Uncharacterized protein	*Moraxella lacunata*	0.23	11.3	4.64E+0	30-333

Bold indicates structures used in alignment in **Supp Figure 4**.

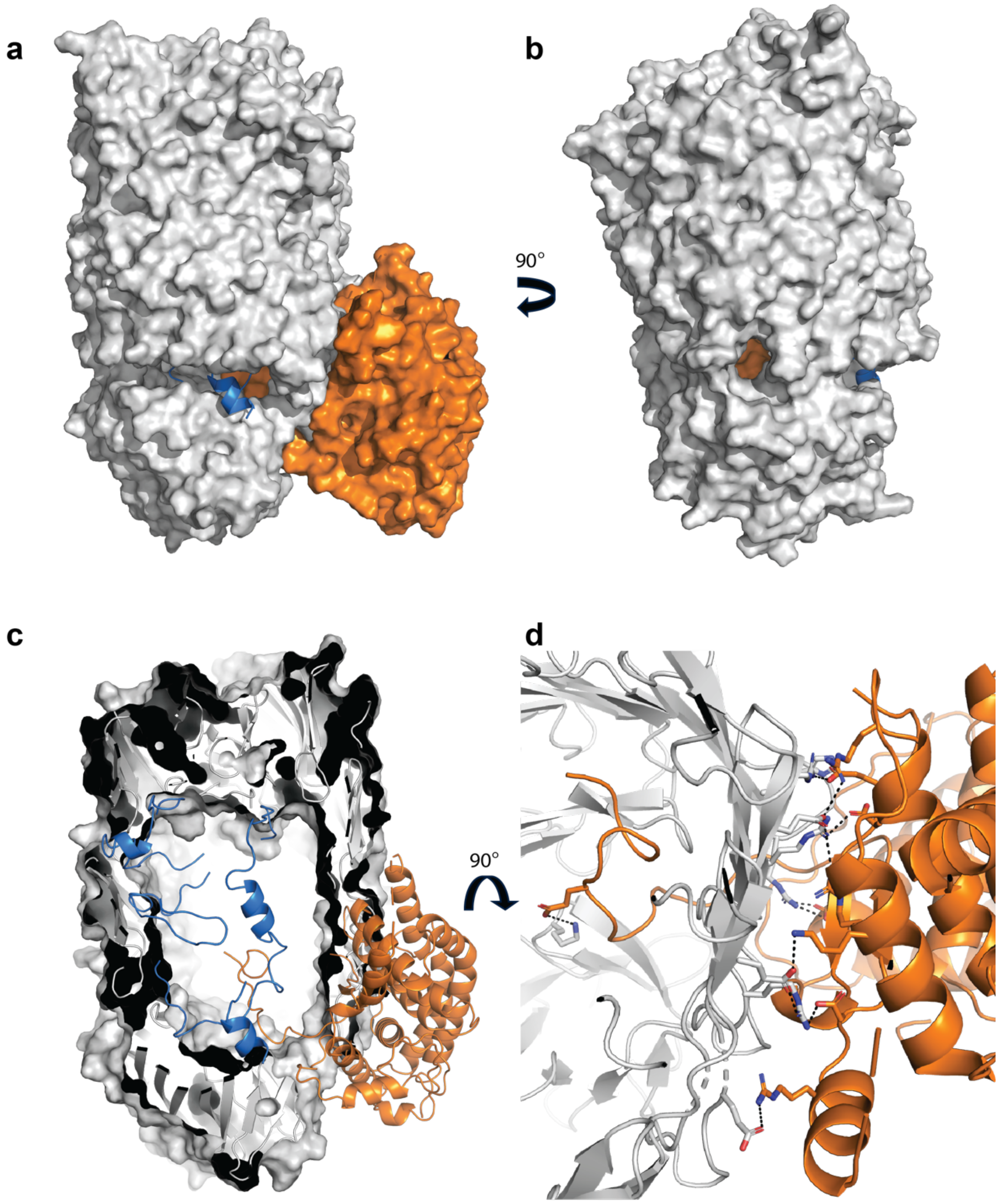

Supp Figure 5. Access to the interior of the Rhs cage and interactions with the clade domain. Tse15 is shown in surface fill with the Rhs domain in grey and clade in orange. The toxin is shown in cartoon with carbon atoms coloured blue. **a**) and **b**) Access to the interior of the Rhs cage occurs via an opening between the bottom and middle β-stranded substructures. The toxin can be visualised in the opening. **c**) Through the opening, the clade domain can be visualised to be well-coordinated with the Rhs domain, as well as close to the toxin density. **d**) Salt bridges dominate the interface of the Rhs:clade interaction where both are shown in cartoon, with the Rhs domain in grey and clade in orange. Residues forming salt-bridges are shown as sticks and coloured by atom.

Supp Table 3: Interactions between the clade and Rhs domains in Tse15.
Generated by PDBePISA[2].

	Clade residue [atom]	Distance (Å)	Rhs residue [atom]
**Salt bridges**			
1	GLU18 [OE1]	2.81	HIS589 [NE2]
2	GLU18 [OE2]	2.93	ARG619 [NH2]
3	ASP32 [OD1]	2.78	ARG593 [NH2]
4	ASP32 [OD1]	2.90	ARG593 [NH1]
5	ASP32 [OD2]	3.04	ARG593 [NH2]
6	GLU127 [OE1]	2.91	ARG617 [NH1]
7	GLU127 [OE1]	3.30	ARG617 [NH2]
8	ASP308 [OD2]	2.49	ARG638 [NE]
9	ASP308 [OD2]	2.84	ARG638 [NH2]
10	GLU326 [OE2]	3.94	LYS400 [NZ]
11	ARG131 [NH1]	3.47	GLU625 [OE2]
12	LYS142 [NZ]	3.41	GLU646 [OE2]
13	ARG143 [NH2]	3.76	GLU625 [OE1]
14	ARG303 [NE]	2.90	GLU650 [OE1]
15	ARG303 [NH2]	3.90	GLU650 [OE1]
**Hydrogen bonds**			
1	GLU18 [OE2]	1.78	TYR608 [HH]
2	GLU18 [OE2]	2.04	GLN594 [HE22]
3	GLU18 [OE2]	2.45	ARG619 [HH22]
4	ASP32 [OD1]	2.33	ARG593 [HH22]
5	ASP32 [OD1]	2.47	ARG593 [HH12]
6	GLU127 [OE1]	2.12	ARG617 [HH12]
7	GLU138 [OE2]	2.47	ASN889 [HD21]
8	ASP308 [OD2]	1.83	ARG638 [HE]
9	ASP308 [OD2]	2.26	ARG638 [HH21]
10	ASP308 [OD2]	3.89	SER629 [OG]
11	GLN316 [OE1]	2.48	ARG593 [H]
12	ALA322 [O]	1.93	ARG402 [HE]
13	ALA322 [O]	2.41	ARG402 [HH21]
14	LYS137 [HZ1]	1.63	PHE642[O]
15	LYS142 [HZ1]	2.29	GLY644 [O]
16	ARG303 [HE]	2.26	GLU650 [OE1]
17	GLN316 [H]	2.22	ARG591 [O]
18	LEU320 [H]	2.02	LEU611 [O]
19	SER332[H]	2.23	ILE336[O]

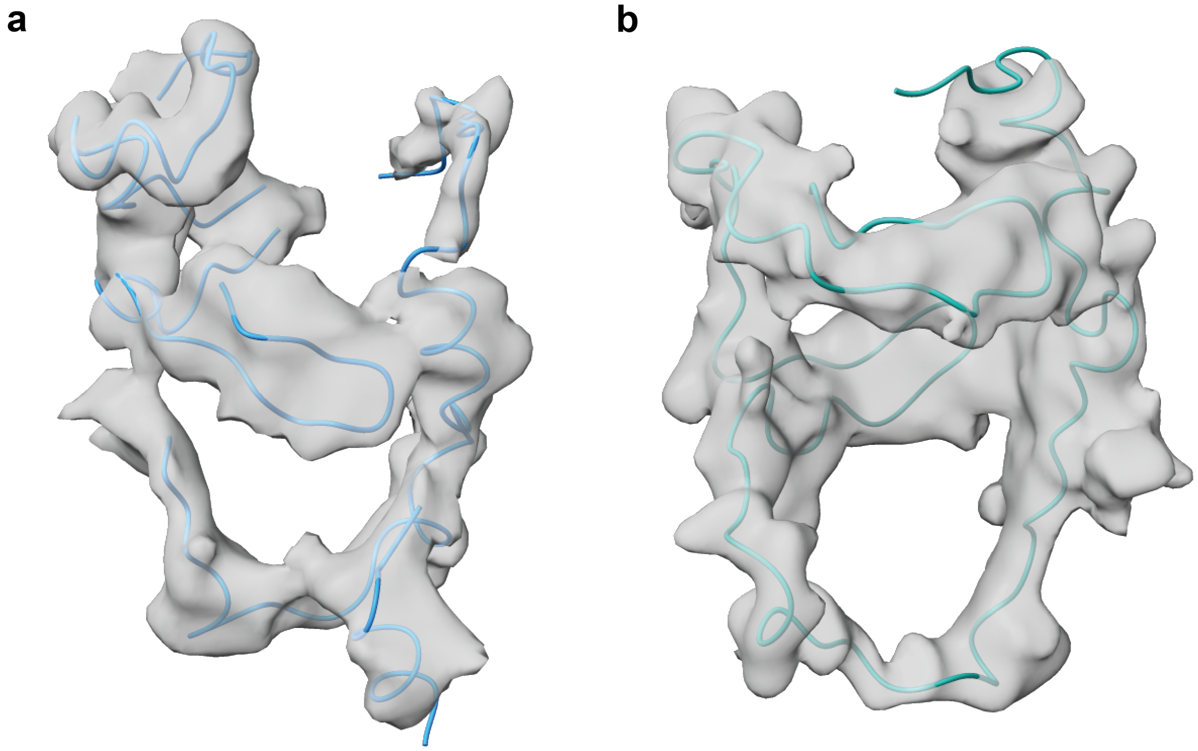

Supp Figure 6. Toxin difference maps of Tse15 and Tse15$_{NN}$ with fitted toxin residues. Toxin density for both maps is shown in grey and is set to show density around the chains at a range of 2.5 on ChimeraX. **a)** Tse15 with the toxin shown in blue, map set at an RMSD of 9.7 Å and **b)** Tse15$_{NN}$ with the toxin shown in cyan, map set at an RMSD of 5.1 Å.

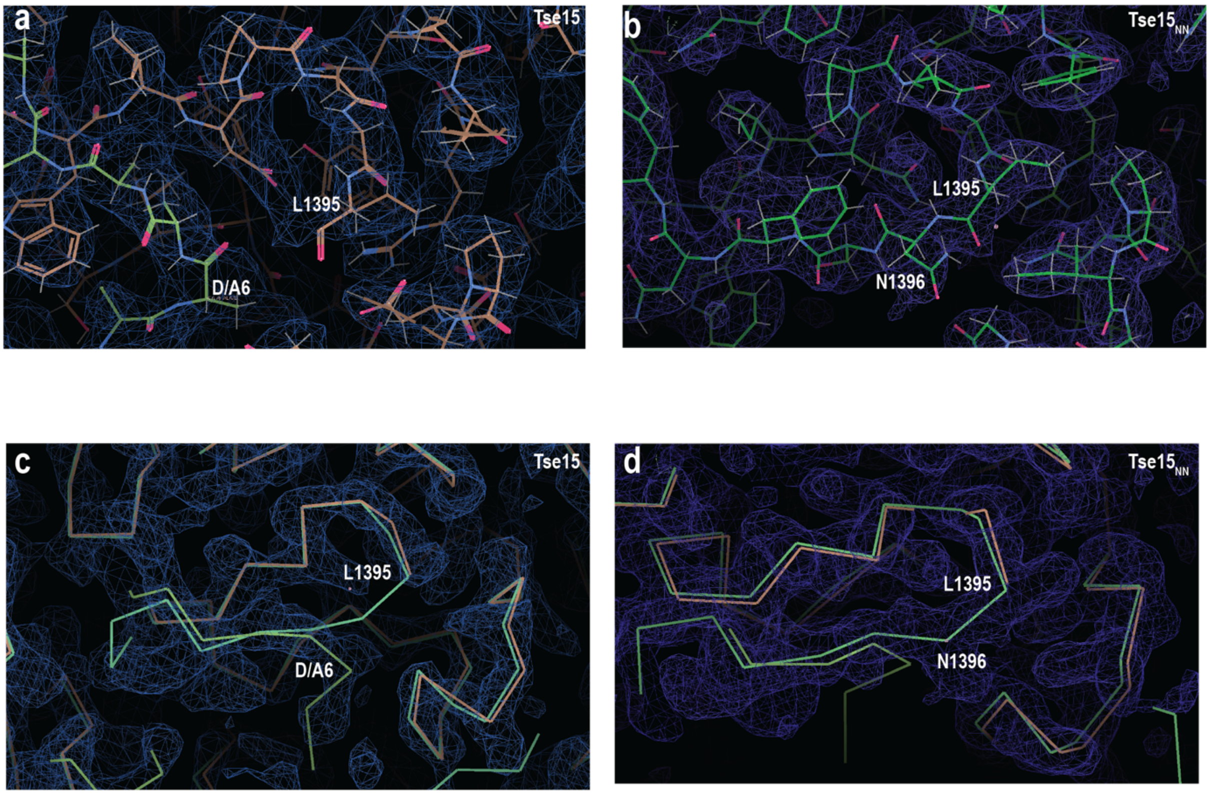

Supp Figure 7. Density of the toxin cleavage site in Tse15 and Tse15$_{NN}$. Carbon atoms show Tse15 in bone (chain A) and green (chain D); Tse15$_{NN}$ (chain A) in dark green. Coulomb potential maps for Tse15 (**a, c**; r.m.s.d. contour 6.0) and Tse15$_{NN}$ (**b, d**; r.m.s.d. contour 5.0) are shown in blue. The position of toxin autocleavage, L1395 is indicated as is the next residue N1396 for Tse15$_{NN}$. The position of the toxin peptide from the chain D of Tse15 is indicated as A6.

a

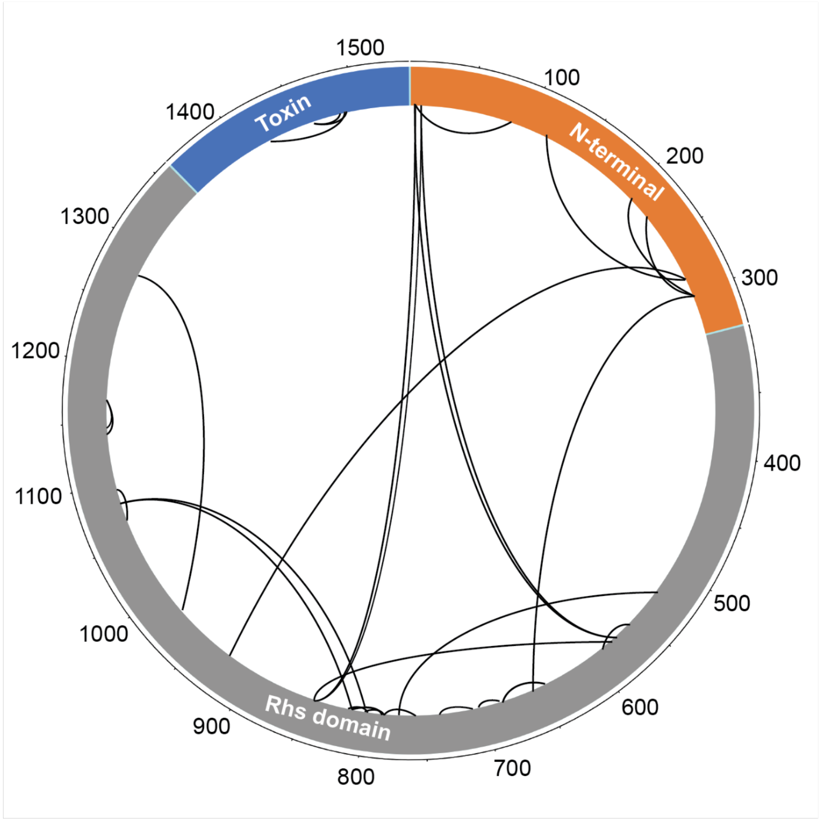

b

Crosslinked residues	
3	846
3	587
9	85
9	587
9	846
137	288
210	306
225	306
288	925
306	659
512	784
569	587
571	587
585	846
659	682
690	701
713	732
771	784
780	797
780	810
797	810
797	1077
810	784/797
815	1077
971	1279
1072	1091
1140	1166
1145	1166
1434	1491
1471	1486
1471	1491

Supp Figure 8. Crosslinking mass spectrometry of Tse15. For both **a)** and **b)**, orange indicates the N-terminal domain, grey the Rhs domain and blue the toxin domain. **a)** crosslinks are mapped onto the Tse15 sequence. Residue numbers are indicated on the outside, domains are coloured and labelled. **b)** crosslinks in list form for clarity

a

```
Tse15    HSARYVSKDPIGLEGGMNTSSYVSDPNQWIDPKGLNSFNY 1400
Tse15NN  HSARYVSKNPIGLEGGMNTSSYVSDPNQWINPKGLNSFNY 1400
         ********:********************:*********
```

b

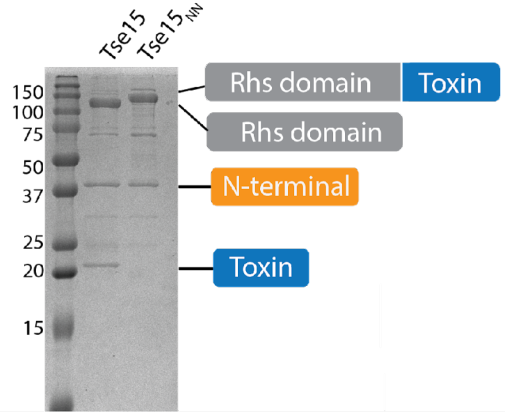

c

Tse15$_{NN}$ Rhs and toxin domain band (92% coverage):

MAWSHPQFEKSAAKENTQQKEIKRQPAVELAVFNLNSVTDVADLQMIASQVQLYLQVCGNTTLEQIKSKANITTVANIFALTGSVLDLMLYATDKKTGDAAVQR**G**
ALLAANLIGLFSEPNNEAHARMALRPMFGLMAECLYRENGKIKETDIKRLGLHLNAM**IAGDLENFLK**ETQAKLSSLLISATTLGVTILQSMATPATGINAGITTA
AGASAEKRDPKLKFTNWAVPLIDLLGK**PSQANLTPKIQPNITSR**LQQEATQAIAALSQTLQQQANAGQK**YTLAWLLQETLK**AIQALENK**GNASVPINQTGEYER**H
TKGDTLEFVSLQADALNAPPCEGADSQSGK**SISYSIGAERVQHADFYLPKIGFSFIRQYNSQMDEFDQSMVGARWMMPFSNMIQQNAQGYLFIDSK**GR**KHQLPVS**
IIFETYEVPYEGWIIKPLKNGELILDFGGEWRSHFQSFDGGKNYYLVKKMNETSQEEILLEYLLLDHIAYLKVINFK**LKQAEYELKFAFNEQVKIIAVFLDDKAE**
PLARYEYDTQGNLIKAIDQNGHTRTYEYNQFHQLTRYTDRTGRGQN**IRYESTEAK**AK**AIEEWADDGSFHTKLK**WHPRLR**QVAVYDAYDVPTYYYFDLDGFTYR**TR
LADGR**ESWYSRDGK**KRITR**QIDFDGRETQQEYNDQDQLVKIVQPNGGIIR**FAYNK**QGNLVEIKDPEGSIWKREYDENRNVSKEINPLGHITQYKYNNDNQLVEVI**
DAKGGVKKIQYNELGQMISYTDCSGKSSTWEYDEDGALTAEQTANNKVVQYFYSTKGR**DKGQLQSIIYPDGLKEYFEHDEEGRLLKHTDTKGLVTEYKYNQVGLL**
EQRIDANR**HSVAYQWDKQGR**IQK**LINQNQAEYLFGYNPYGYLIREQAFDGEEKHYSYNENGR**LFQI**RRPNILTQFDYYADGQIASKSFTHLHTGQKQTEQFDYNL**
NSQLSRASNEVSQIDLYRNALGQLVREHQHYKIPELKPLTAVLHYEYDELGNLIKTIRPDGHTLNHLVYGSGHIYAIGLNNQEVVSFQRDDLHRETTR**LLANGLM**
QTKQYNDVGLLSSQFIQPEQETQDYLQYQAHRKYHYDKNYLLSQVEDSRLGK**LNYQYDPIGRLIAAQSLHKTESFNFDPAGNLIDSESVLSPAQIKNNLIKSYKG**
KHYQYDVQGNVTEIIQAGKNLK**LTWDNQNR**LIR**SDNNGLVTEYGYDVFGR**RLYKKTAK**ELTLFGWDGDLMIWESFKSAQTNYTKHYIYEPDSFVPLLQAGYKDFI**
QLIETPDYQEYQTKPYSIYKDPVWNRNLGKER**TALEQFTFYHCDQVGTPQTMTNIRGECVWEILQDTWGAVSQIKALNQDNPFEQNNLRFQGQYYDRETELHYNR**
YR**YYEPHSAR**YVSK**NPIGLEGGMNTSSYVSDPNQWINPKGLNSFNYGEMFGIPASAQSGLAYQGQRNYECYAETGELCKIKVPPLFDYVACSGGGLGIGVGFVKN**
QWTGEYYISGSKDSLLIPVAKSVAQNKQFSAKDLAGASCVGGNIHNIPSYTKTTMTMGEITNEFVSGASVTVGGGAYGAVANVVVPLVSKSSPVKGTWASELGVG
TPGFNVGVSGTVSVDTILDAVKPSKKHHHHHH

Supp Figure 9. Tse15$_{NN}$ construct design and purification. a) sequence alignment showing mutation site for Tse15$_{NN}$. Mutation sites are indicated in red, while blue indicates the start of the toxin domain. **b)** Coomassie stained SDS-PAGE gel showing purified Tse15$_{NN}$ compared to Tse15 wild-type. Domains as determined by mass spectrometry are shown. **c)** Mass spectrometry peptide fingerprinting analysis of the Tse15$_{NN}$ Rhs and toxin domain band excised from SDS-PAGE. Protein domains are coloured as follows: N-terminal clade residues orange, Rhs domain residues grey and toxin residues blue. Percentage peptide coverage is indicated at the top. Residues shown in bold indicate peptides identified.

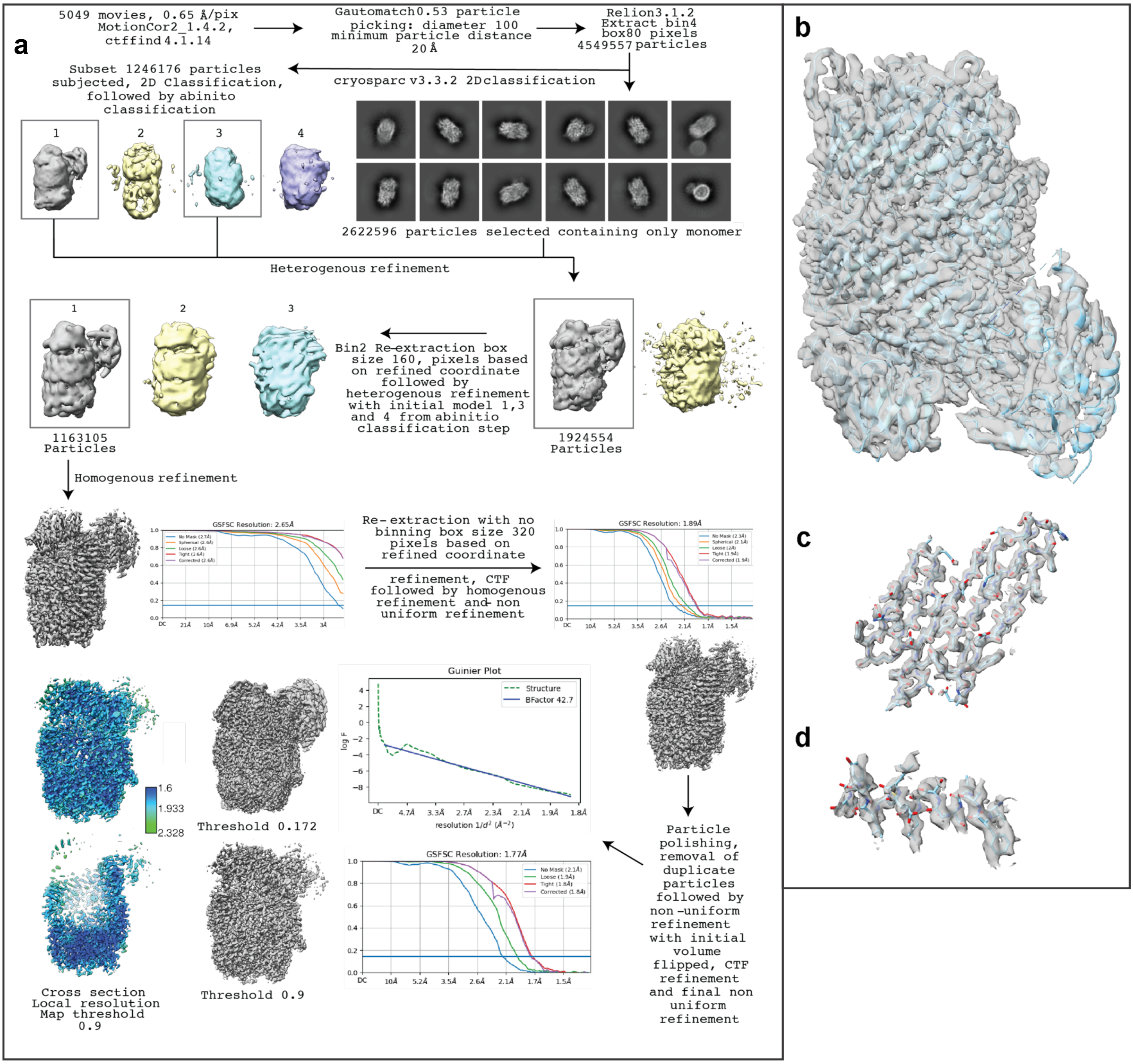

Supp Figure 10. Workflow for solving Tse15$_{NN}$ structure and map to model fit. (Left box, a). Particles were picked using Gautomatch, then extracted using Relion. Cryosparc was used for 2D classification, and four different *ab initio* models were produced. Heterogenous refinement was conducted using *ab initio* maps 1 and 3. Particles were re-extracted using a larger box size, and heterogenous refinement was conducted using initial models 1, 3 and 4 from the *ab initio* reconstruction. Homogenous refinement of model 1 was used to produce a map at 2.65 Å, particles were then re-extracted based on refined coordinate refinement, CTF refinement, followed by both heterogenous refinement and non-uniform refinement to produce a map domain at 1.89 Å. Particles were polished, duplicate particles removed and then non-uniform refinement was conducted alongside further CTF refinement and non-uniform refinement to produce a final model at 1.77 Å. Final maps shown at a threshold of 0.172 and 0.9. Local resolution is also shown at a map threshold of 0.9. **Right box** shows map to model fit **(b)** Tse15$_{NN}$ fit to model shown as cartoon (contour 0.15); (**c**) Rhs domain β-sheet residues 900 – 952 (contour 1.0) and (**d**) clade domain α-helix residues 67 – 82 (contour 0.3). Both **c)** and **d)** are shown as sticks and coloured by atom.

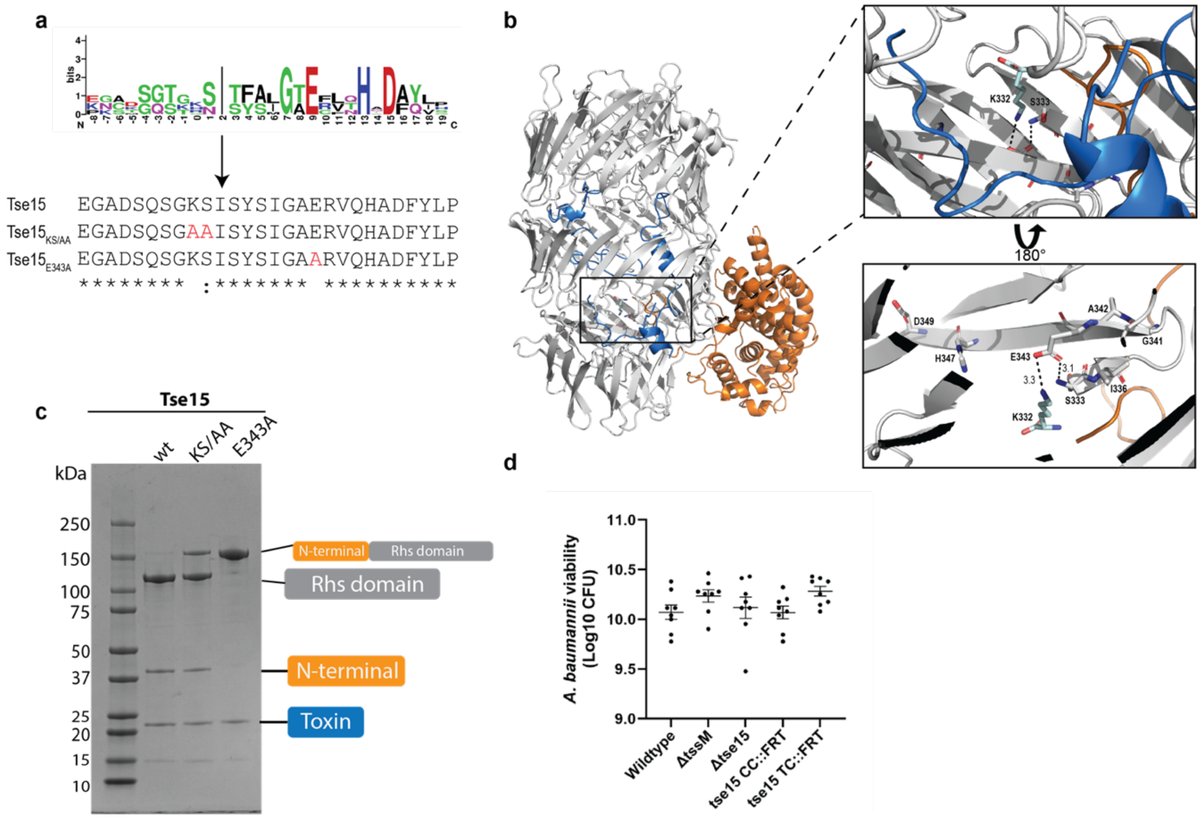

Supp Figure 11. Identification and mutation of the clade autocleavage motif. a) *Acinetobacter spp* N-terminal cleavage site consensus shown as a sequence logo. Residues are coloured by property. Below the consensus is a sequence alignment of the N-terminal cleavage site in Tse15 and positions of KS and E343A mutations introduced. **b**) Location of the cleavage motif shown on the structure of Tse15 coloured by domain, clade orange, Rhs grey and toxin blue. The inset zoom shows the motif residues shown as sticks with K332 provided by the AlphaFold2 model and shown in cyan carbon stick. Below box is the same structure rotated 180° to show position of motif residues and interactions of nucleophilic E343. **c**) Coomassie stained SDS-PAGE showing N-terminal cleavage mutations, boxes to the right indicate domains. **d.** *A. baumannii* predator viability following T6SS competitive killing assays to determine the effect of replacing the Tse15 clade cleavage motif or the toxin cleavage motif with an in-frame FRT site on the ability of *A. baumannii* to kill vulnerable *E. coli* prey in a Tse15-dependent manner. *A. baumannii* predator strains used were AB307_0294 wild-type, a *tssM* deletion mutant (inactive T6SS), a *tse15* deletion mutant, a *tse15* clade cleavage mutant (Tse15CC::FRT) and a *tse15* toxin cleavage mutant (Tse15TC::FRT). Bars represent mean of four biological replicates, error bars represent SEM.

Supp Table 4. Conservation of the clade autocleavage motif across N-terminal homologues. Searching FoldSeek[1] for homologous sequences identified that the autocleavage motif is highly conserved. The motif extent within Tse15 is shown in bold with conserved motif residues shown in red. Sequences above the black line had a probability score of ≥ 0.84. Sequences below the line did not show the motif but had probability scores of ≤ 0.35.

**Tse15**	**GKSISYSIGAERVQHADFY-LP-KIGFSFIRQYN----SQMDEFD-QSMV----------**	**375**
Paraburkholderia_steynii	GGSINYVMGDENLEQTDFA-LDGVVPIVWTRLYR----SSLVAYD-GSVL----------	407
*Acinetobacter_bereziniae*_LMG_1003	EHSISYAIGAERVNHADFY-LP-KLGFAFSRQYN----SQMNEFD-YSMI----------	388
*Acinetobacter*_sp._WC-743	--	172
*Acinetobacter*_sp._DSM_11652	AHSISYSIGAERVSHVDFS-LP-KLGLVFNRLYN----SQMAEFD-NGAL----------	379
*Acinetobacter*_sp._OIFC021	GKSISYSIGAEWVQHADFY-LP-KIGFSFIRQYN----SQMDEFD-QSMV----------	375
*Acinetobacter_baumannii*_b	GKSISYSIGAERVQHADFY-LP-KIGFSFTRQYN----SQMDEFD-QSMV----------	375
*Acinetobacter_baumannii*_c	GKSISYSIGAERVQHADFY-LP-KIGFSFIRQYN----SQMDEFD-QSMV----------	290
*Acinetobacter_baumannii*_a	GKSISYSIGAERVQHADFY-LP-KIGFSFIRQYN----SQMDEFD-QSMV----------	375
Variovorax_paradoxus	--	328
Hydromonas_duriensis	TCDINFAMGVERVFHEDFS-IGHVLPIEWVRTYS----SAYAQYD-QSVM----------	444
*Variovorax*_sp._CF313_a	GGAISLATGCESFGHTDFV-LGAPLPITWTRTYR----SDLAAFD-QGSL----------	393
*Variovorax*_sp._679	GGSISLVTGCESFTHTDFV-LEAPLPIEWARTYR----SQLDAYD-RGVL----------	298
*Variovorax*_sp._CF313_b	GASISLSTGNESFTHTDFV-LAAPLPIEWARTYA----SDLDAFD-RSSL----------	293
*Variovorax*_sp._YR750_a	RFAISLATGCESFTHVDFA-LVAPMPITWARTYR----SNLGAFD-EGSL----------	414
*Variovorax*_sp._OK202	GASISLATGNESFMHTDFV-LA--------------------------------------	375
*Variovorax*_sp._YR750_b	--	315
*Pseudomonas*_sp._VLB120	GRTVHYLYEPGSFVPVAQALRKRPIVLHRQPDWR----QREYDFD-QDPLWQTTMPPQAF	370
*Acinetobacter_baumannii*_d	KRNITFALGTECLTHQDAY-VSSILSFVLIRKYA----SNLYQLD-HGEF----------	416
*Acinetobacter_baumannii*_118362	GGSITFAMGTEFFTHVDAQ-LGGIIQDSISRTYV----SNLYQMD-DAIF----------	404
*Acinetobacter_baumannii*_e	GGSITFAMGTEFFTHVDAQ-LGGIIQDSISRTYV----SNLYQMD-DAIF----------	405
*Acinetobacter*_sp._a	KRSITFAMGTEFLNHTDAQ-IHPLLIEQFSRTYV----SNLYQYD-QSIF----------	392
*Acinetobacter_guillouiae*_NIPH_991	SGSISFAMGTEFLTHDDAL-IHPLMVEPLARSYI----SNLYQYD-QSIF----------	392
Trinickia_fusca	PRSVGFALGDERIEHEDFV-LEGPLPIVWQRTYR----SFFDANDAHGEL----------	416
Burkholderia_contaminans	IGSISYSFGDETFSHADFE-LPGAMPVVWMRVYR----SRLAGYD-DGEL----------	374
*Burkholderia*_sp._Bp9004_c	TGSIDFAFGDETFTHTDFE-LPGALPLVWARTYR----SRLSAYD-TREL----------	398
*Burkholderia*_sp._Bp9142	KASIDFAFGDETFTHTDFD-LPGALPLVWARTYR----SRLSAYD-NGEL----------	398
Burkholderia_cenocepacia	EASIDFAFGDETFSHVDFD-LPGAMPLVWERTYR----SRLSAYD-NGEL----------	399
*Burkholderia*_sp._4701	--	330
*Burkholderia*_sp._AU6039	IGSISFARGAERIDH---	374
Burkholderia_stagnalis	GRAIGLALGDESFTHTDFA-LPGVVPVEWARTYR----SNFGAHDEQGPL----------	407
Burkholderia_reimsis	GGAIGLALGDESFTHTDFV-IPGVMP----------------------------------	386
Paraburkholderia_rhizosphaerae	TGSIGFALGDESFTHTDFV-LPGVLAVEWVRAYR----SNFDAHDTAGPL----------	405
Pseudomonas_putida	NCSISFATGSETIVHTDFQ-LPGAFPIEWYRTYR----STLSAFD-DSPY----------	367
*Burkholderia*_sp._Bp9004_d	--	314
*Burkholderia*_sp._Bp9004_b	GS--	317
*Burkholderia*_sp._Bp9004_a	GSNISFATGSESLTHTDFI-LPGPFPIAWTRSYR----SSQAAYD-SGEL----------	389
Burkholderia_cepacia	GGSISFATGTETLSHTDFV-LPGPFPIVWTRTYR----SSLGAYD-AGEL----------	390
*Pseudomonas*_sp._LP_8_YM	GSNISFATGSESFTHTDFS-LPGCFPIEWARTYR----SSLSALD-EGPL----------	361
*Pseudomonas*_sp._FSL	GCSISFATGSETVAHADFS-LPGPFPVEWVRTYR----SSLDALD-HGSL----------	362
*Pseudomonas*_sp._URIL14HWK12:I1	CNRISFARGSEQITHTDFT-LPGPFPVTWTRNYR----SSLSALD-TGPL----------	375
*Pseudomonas*_sp._FSL_R10-0765	GHSISFARGSEYLAHTDFT-LAGPFPIEWSRAYR----SSQSALD-SGAF----------	371
*Pseudomonas*_sp._c	GHSISFARGSEYLAHTDFT-LAGPFPIEWSRAYR----SSQSALD-NGVF----------	371
Pseudomonas_helleri	GHSISFARGSEYLAHTDFT-LAGPFPIEWSRAYR----SSQSALD-SGVF----------	371
*Pseudomonas_helleri*_b	GHSISFARGSEYLAHTDFT-LAGPFPIEWSRAYR----SSQSALD-SGVF----------	371
Andreprevotia_lacus	KNSIDFAMGTESVSHVDFV-LNGVFPLQWSRTYR----SNLAAFN-DSEQ----------	398
Pseudomonas_lundensis	CNSILFSLGAETFSHTDFS-LPGPFPIDWTRT----------------------------	367
Pseudomonas_graminis	IKSISFAMGSESVSHTDFS-LPGPFPIEWTRTYC----SSLDAYD-QDII----------	406
*Pseudomonas*_sp._a	IGSISFAMGSESVSHTDFS-LPGPFPIEWTRTYC----SSLDAFD-EDVV----------	400
*Pseudomonas*_sp._b	GNSISFAMGSESVRHTDFS-LPGPFPIEWARTYC----SSLDAYD-QDVI----------	400
Pseudomonas_cichorii	CNSISFALGSETFSHTDFS-LPGPFPIHWARLYN----SRLSAYD-QGIM----------	394
Pseudomonas_sp._WS_5354	--	*0*
Pseudomonas_helmanticensis	--	*0*
*Neisseria*_sp._HMSC065C04	GRAVAVQKTKGAVEQISNK-ITGLIGEHMADYWMLEQVGGKARHDHGGTA----------	368
Xanthomonas_sacchari	PLHAAVQVTRKAYQNIAKR-QKGLIGEHMADYHELKRLGGHWPHDNAKGHWS--------	328

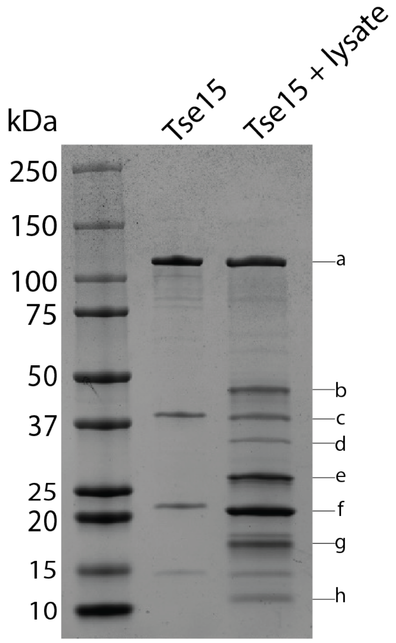

Band	Protein identified	# of unique peptides	Coverage of full-length protein (%)
**a**	**Tse15 Rhs domain**<br>**VgrG15**	**381**<br>**142**	**58**<br>**80**
b	Homoserine dehydrogenase	23	41
c	Tse15 clade domain	224	46
d	Truncated Tse15 clade domain	20	17
e	Nucleotide-binding protein J522	87	61
f	Inorganic pyrophosphatase<br>Tse15 toxin domain	101<br>71	93<br>20
g	30S ribosomal protein	17	52
h	50S ribosomal protein	10	50

Supp Figure 12. Pulldown of native VgrG15 with recombinant Tse15. Left shows an SDS-PAGE gel of Tse15 alone and following incubation with lysate from *A. baumannii* A307_2094 harbouring pAL1415 (VgrG 15 expression construct). Tse15 and all proteins that were pulled out by Tse15 or the nickel resin, are labelled a – h. **Right** shows the identity of each band as determined by mass spectrometry-based peptide fingerprinting. The number of unique peptides, and total coverage of the full-length protein are as indicated.

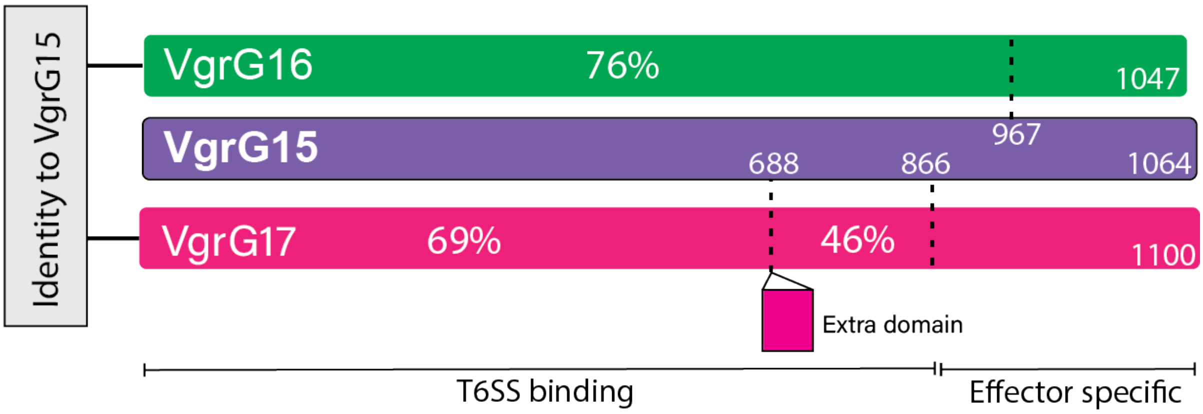

Supp Figure 13. The VgrG15 N-terminal domain is highly conserved as compared to the N-terminal domains of the other *A. baumannii* AB307-0294 VgrG proteins. Green indicates VgrG16, purple VgrG15 and pink VgrG17. The amino acid length of each is indicated by the number at the end of each schematic. Percentages show sequence identity between either VgrG16 or VgrG17 and VgrG15 in the regions between where the dashed lines are placed. The small pink box below VgrG17 denotes an extra domain present in VgrG17 that is not found within the other VgrGs. Overall, we predict that amino acids required for overall T6SS structure are within the N-terminal region, while the C-terminal region is specific for interaction with the effectors.

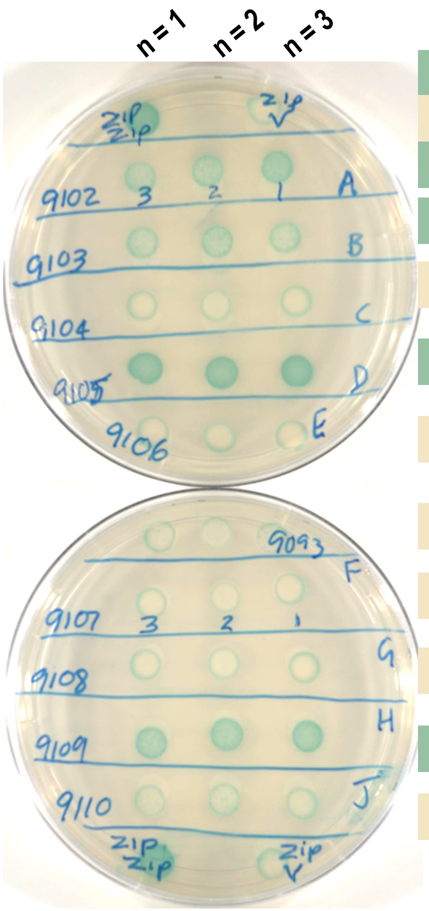

Zip/Zip	T25_zip + 18C_zip (positive control)
Zip/V	T25_zip + no protein (pUT18C vector) (negative control)
9102	T25_Tse15$_{2\text{-}1389}$+ 18C_V995A S996A VgrG15$_{976\text{-}1014}$
9103	T25_Tse15$_{2\text{-}1389}$+ 18C_K997A Q998A VgrG15$_{976\text{-}1014}$
9104	T25_Tse15$_{2\text{-}1389}$+ 18C_L999A N1000A VgrG15$_{976\text{-}1014}$
9105	T25_Tse15$_{2\text{-}1389}$+ 18C_L1001A N1002A VgrG15$_{976\text{-}1014}$
9106	T25_Tse15$_{2\text{-}1389}$+ 18C_ L1003A S1004A VgrG15$_{976\text{-}1014}$
9093	T25_Tse15$_{2\text{-}1389}$+ 18C_ L1011A G1012A VgrG15$_{976\text{-}1014}$
9107	T25_Tse15$_{2\text{-}1389}$+ 18C_ D1006A Q1007A VgrG15$_{976\text{-}1014}$
9108	T25_Tse15$_{2\text{-}1389}$+ 18C_ M1008A VgrG15$_{976\text{-}1014}$
9109	T25_Tse15$_{2\text{-}1389}$+ 18C_G1012A VgrG15$_{976\text{-}1014}$
9110	T25_Tse15$_{2\text{-}1389}$+ 18C_L1011A VgrG15$_{976\text{-}1014}$

Supp Figure 14. Example of one two-hybrid experiment showing selected alanine substitutions. The colour of the *E. coli* colonies on the plate were used to visually score the interaction between the proteins expressed. A blue/green colour throughout entire colony was deemed as a positive interaction (see Zip/Zip example of positive control: top plate, top left) and a cream colour with blue/green edge was considered negative (see Zip/V example of negative control: top plate, top right). On the left are photographs of the agar plates and on the right is the legend for which protein interaction was being assayed. There are three colonies per line represents the three biological replicates for each interaction (also indicated by n=1, 2, 3 at the top of the image).

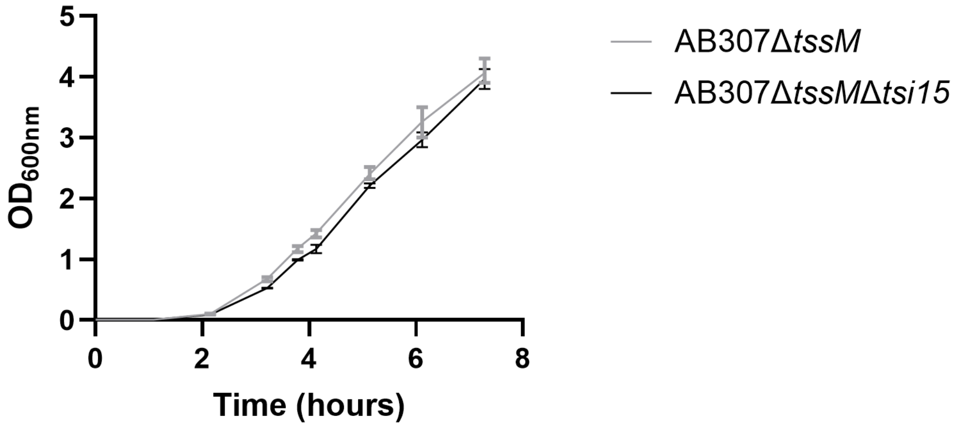

Supp Figure 15. Tsi15 is not required to protect the host cell. Growth of *A. baumannii* AB307 mutant strains measuring OD_{600} over time. Relative growth of *A. baumannii* Δ*tssM* mutant (unable to produce an active T6SS) and the *A. baumannii* Δ*tssM/tsi15* double mutant that cannot produce the Tse15 cognate immunity protein, Tsi15, or export the effector Tse15 via the T6SS. Error bars represent the standard error of the mean (n=3).

Tse15

MAKENTQQKEIKRQPAVELAVFNLNSVTDVADLQMIASQVQLYLQVCGNTTLEQIKSKANITTVANIFALTGSVLDLMLYATDKKTGDAAVQR
GALLAANLIGLFSEPNNEAHARMAIRPMFGLMAECLYRENGKIKETDIKR**LGLHLNAMIAGDLENFLK**ETQAKLSSLLISATTLGVTILQSMA
TPATGINAGITTAAGASAEKRDPKLK**FTNWAVPLIDLLGKPSQANLTPKIQPNITSRLQQEATQAIAALSQTLQQQANAGQKYTIAWLLQETL**
KAIQALENKGNASVPINQTGEYERHTK**GDTLEFVSLQADALNAPPCEGADSQSGK**SISYSIGAERVQHADFYLPKIGFSFIRQYNSQMDEFDQ
SMVGARWMMPFSNMIQQNAQGYLFIDSKGRKHQLPVSIIFETYEVPYEGWIIKPLKNGELILDFGGEWRSHFQSFDGGKNYYLVKKMNETSQE
EILLEYLLLDHIAYLKVINFKLKQAEYELKFAFNEQVKIIAVFLDDKAEPLAR**YEYDTQGNLIK**AIDQNGHTRTYEYNQFHQLTRYTDRTGRG
QNIRYESTEAKAKAIEEWADDGSFHTKLKWHPRLRQVAVYDAYDVPTYYYFDLDGFTYRTRLADGRESWYSRDGKKRITR**QIDFDGR**ETQQEY
NDQDQLVKIVQPNGGIIRFAYNKQGNLVEIKDPEGSIWKREYDENRNVSKEINPLGHITQYKYNNDNQLVEVIDAKGGVKKIQYNELGQMISY
TDCSGKSSTWEYDEDGALTAEQTANNKVVQYFYSTKGRDKGQLQSIIYPDGLKEYFEHDEEGRLLKHTDTKGLVTEYKYNQVGLLEQRIDANR
HSVAYQWDKQGRIQKLINQNQAEYLFGYNPYGYLIREQAFDGEEKHYSYNENGRLFQIRRPNILTQFDYYADGQIASKSFTHLHTGQKQTEQF
DYNLNSQLSRASNEVSQIDLYRNALGQLVREHQHYKIPELKPLTAVLHYEYDELGNLIKTIRPDGHTLNHLVYGSGHIYAIGLNNQEVVSFQR
DDLHRETTRLLANGLMQTKQYNDVGLLSSQFIQPEQETQDYLQYQAHRKYHYDKNYLLSQVEDSRLGKLNYQYDPIGRLIAAQSLHKTESFNF
DPAGNLIDSESVLSPAQIKNNLIKSYKGKHYQYDVQGNVTEIIQAGKNLKLTWDNQNRLIRSDNNGLVTEYGYDVFGRRLYKKTAKELTLFGW
DGDLMIWESFKSAQTNYTKHYIYEPDSFVPLLQAGYKDFIQLIETPDYQEYQTKPYSIYKDPVWNRNLGKERTALEQFTFYHCDQVGTPQTMT
NIRGECVWEILQDTWGAVSQIKALNQDNPFEQNNLRFQGQYYDRETELHYNRYRYYEPHSARYVSKDPIGLEGGMNTSSYVSDPNQWIDPKGL
NSFNYGEMFGIPASAQSGLAYQGQR**NYECYAETGELCK**IKVPPLFDYVACSGGGLGIGVGFVK**NQWTGEYYISGSKDSLLIPVAK**SVAQNKQF
SAK**DLAGASCVGGNIHNIPSYTK**TTMTMGEITNEFVSGASVTVGGGAYGAVANVVVPLVSKSSPVKGTWASELGVGTPGFNVGVSGTVSVDTI
LDAVKPSKK

VgrG15

MFNNIFQILESFGFLSQHR**SVYLQFSDASLNSQVFLQRIDGQHYLNQGMTAELICLSTNAHIPLKTFIGLQVAVDQVTDR**GSFFR**TTGIITGA**
SQGQSDGALTLYKLTVSDPTYLWHKRRNSRVFMNKSVK**EISEILFQEWQGKSPLFASSLTLDLSGLKQTYDVRPFVMQLNESDYDFLTR**LWR**S**
EGISWLIDEAELTVASNMDNIQPQKLR**LIDDNNQYQALTR**RVIRYHR**SSATEQFDSMTSLMADRSLQPTSIFVQRWQPDVLQQTDGAGSVQSK**
HQHSTNYDNQSLSLEEAWHFSPAWMQDLNGEDGATSASNQQIEKFNQNLSAYYDAQSKQFIAKTTVR**DTQVGYWFELNEHPEIDQHESTDKEF**
LIIGKNYYNQNNLPKDLNQQIQTLLQQSDWQASNTDERQANQLILQRR**YIPTTPAYNPQTHSPVAHPQR**AK**VVGPEGEEIYVDEWGR**IKVRFL
FTR**SDDHSHDGGAGTNNNDTDSAWIDVLTPWAGEGYGAR**FLPRIGEIVVIDFFNGDIDRPFVMGRIHEAQRHPTKFDNKGKLPDTKKLSGIRS
K**EVSGSGFGQLRFDDTPGQISTQLQSSHGASQLNLGK**LSHPK**DKAESEDRGEGFELRTDQWGALRAGQGLLVSTHK**QDNAK**GDHLDAEVAK**KQ
LEGSQTNSKALSDIAK**NQKTDEIESIEQLKDFASQIQQQIAK**FEKALLLLSSPDGIALSSSEDIHISADAQINQIAGDSINISTQK**NVIAHAQ**
NRLSLFAAQSGLKAVAAQGK**VEIQAQADALDVLSKLGITISSTDDKVIISSPK**EVK**ITGGSSQITLNGSGIFPK**TGGK**FQVNAGQHLFMGGAS**
ANASAPELPKAKPMQGALELLRSYGGDNFFKQNSYKVIDSLGKQITGK**LDGNGFAQVTGIAPGPAK**VVFEK**DNTSAWLQSSDFKRNYTWAEPV**
KSVQGLMK**NALEAVGQNTMSQLQNNLLSTDKNSFK**NLGK**NTLDNLAGQTVAQIKNQVTNTALNTVSKQLNLNLSADQMK**SLGQMATNPSQSLE
MLK**EQGGDFLSDQMTAK**LFK**TTNQESPIQQGDLDTFVR**SKK

Tde16

M**SQNSVVAPLNTFSPK**DLTAKKAEVDKWFR**EYTNGVVTVDR**LETICRSVPVLGSAFAIGDIIIDIISMINKGGLDKVEIFDWLNLGIDVIGLV
PMGPVGPSVRSAARPALFYVKNESEKIIKAQAKKLGKKTLTSQEVKKALSTGFKDSASVFLTTIIAENVAGTLENFAKKGQSLLNQILKEVGN
WIVLLTKTIDDGFKKLVSGSLNGLPNLKRAGQQSLGVIK**GIFELDGTR**IVNNAKYATENVAKTVGK**GYVNLANLAVSDEAR**AKVLALGAKIRS
IGQVAQAKVNGLSDPNTLWTIGWLFSIMGMVAAKHRQKRAQIKAKETTKANASHPSTATDKSNKQAHAENNANQCKNCMGGTGGSITFAMGTE
FFTHVDAQLGGIIQDSISRTYVSNLYQMDDAIFGARWVTPFTTKISRKFKYTSKKKDHKDYLNGLEYICLDGRAIDLPDLKKGQSIYDPIEQY
TYTVLSDQLHLIAYGEDEKRYYEKYGEDYRLSYIERKNGFKVALRYDHVSTDNKTILSDILFKQDDNLLAHLALQLTPQGLVSDIWTIKNGQL
DRVLASYDYDQQGDLVQATNEFAASYYYQYTHHLITRYTDLTHRGMNLKWDGILPTSKAIEEWADNASRASKLEWDKNIRKTTVLDVEGNSTE
HYYDIDGYTYRIVYPDNFEEWFFRDDAKNITLHIAKDGSKTSYTYDERGNVLTTTQDDGATSYFEYDEKNQLTGMVDAEQGRWFKQYDGSGNL
IKEIDPLKHETAYVYNAMGLVTSITDAKGGSKSLKYDDQGNLISYTDCSGKETKWQYDERGRVISIENALNQKVEYFYTELTLENREPIIKGL
PLNAFGQLEKIKHADGTEEHFIHDAEGRLLAHVDPKQNITRYEYDEAGLILSRTDALNHKLKYKWDRLGRLTRLINENGASYQFFYDVASRLV
KEIDFDGKETVYHYDEKSGQLATSIEVASAYGQDLKDRAAPKDRIQQFIFDSMGRLEQRTAGYGHYGLELEEKQTEEFAYDYMGRIIQAKNAQ
SNLQWFYDAAGNLVQEHQQDYKINKTAVWKHQYDEINDRIKTTRPDGQVIDWLTYGSGHVQSLIVNGQDFVSFERDDLHREIARHYANGVSQE
QQYDLAGRLKSQMMLSEHENGYQNQYKRHNNALEQTSQLVQRLYQYDKTGELTAIRDTRRGNIAYKYDPVGRLLEASSKLGKETFSFDPASNI
LDSYHSQKVQSHSQKLDETSYGYNRLVNNVVKEYLDQQYQYDAYGQLIRQKTSQGDLNLEWDVYGRMVKSRNSQYTAEYRYDALGRRIQKWSK
HHHTGQEQNIIYGWDGDTLAYESTEELTKHYIYEKDSFVPMLQAVYLSPIELHQTPDWSDRPYNIHRDPLWKTEKEGKEFDDVWFYHCDHLGT
PQEMTDHTGAIIWKAEYKAWGECKAEEAKSNFFENSEIISNNIRFQGQYFDEETGLHYNRYRYYSPYVGRFVSKDPIGLLGGNNVYVYAKNPI
TWIDSKGLCSTTLNRNLGGVKGDHLQAHHIIPEEIWAK**RKDFLDDIGIGGNRDKAENGVLMPDSEAK**AKQMKR**QLYHCGSHPIYSAGINQK**LG
QIQREFESKKITASQAR**DKVANLQSSMRLVLITPGTKPIR**LS

VgrG16

MLNSIHQVLDSLGISPQKRAIHVQFTSSILNAQVFLQR**IDGVHALNDGLK**AELLCLSTNATIQLK**SFIGVQAAVDIVTER**GELTR**VTGIITHA**
QQGQSDGSLTLYKLTLEDPTALWKYRRNSRVFMNK**SVVEIWEILFK**EWQTKNPLFAASLSLDLSGLTQTYDVR**PFVMQHNESDWNFLTR**LLR**S**
ENISWLIDEAQHIVPSTETPIQAQKLR**LIDANSQYQPLDRK**TIRYHR**SSAVEQYDSMTR**LTAER**SLQPNVMHIQRWQAEILDQEEGIGSVQSK**
HQHSEHYDNATLGLEQAWNYSPAWIGDLKGEDGVTKSGNQQVER**LNQNLNNYYEAQAKRFIAQTTVRDAYVGYYFELNEHPEIDQHESTDRSF**
LIISKSFFNQNNLPKDLNDR**INGLLAQSNWAIQNPENSDER**QANQLILQRRHIPTTPAYSPQIHSPVTHPQRAKVVGPEGEEIYVDEWGRIKV
RFLFTRSDDHSHDGGAGTNNNDTDSAWIDVLTPWAGEGYGVRFLPRIGEIVVIDFFNGDIDRPFVMGRIHEAQRQPTKFDNKGKLPDTKKLSG
IRSK**EVSGGGFGQLRFDDTPGQISTQLQSSHGASQLNMGK**LSHPKDKAESEDRGEGFELRTDQWGALRAGQGLLVSTHKQDNAK**GEHLDAEVA**
KKQLEGSQTNSKALSDIAKNQKTDEIESIEQLKDFASQIQQQIAKFEKALLLLSSPDGIALSSSEDIHISADAQINQIAGDSINISTQKNVIA
HAQNRLSLFAAQSGLKAVAAQGK**VEIQAQSDALDVLANKGITISSTEDCIEISSPK**EIVITGASSQITLNGSGIFPKTGGK**FQVNAGQHVFQG**
GASASVKSSLPPPPKR**AQGVLELLHDYSHGAFVKGAGYTVTDNLGK**VVNGKLDDKGFAR**VSGLATGSVKVLFDPDPRNPWDEASDFKRKVEWP**
NQNDVEGSIGIESGSKGLTETLKNDFK**KLSQLASNVK**SVQQKIESVKKIKDQGAK**ALLPMAMEQVLGGSDGSKLSQSFVSQNSFMPSSQQSNP**
LRRLAQASSDKTAITK**IQSPFETFV**

Supp Figure 16. Peptide signatures of Rhs effectors and their cognate VgrG within the secretome of T6SS active *A. baumannii.* Peptides identified by MS/MS are in bold and underlined. The top shows coverage for Tse15 where the domains are coloured: N-terminal clade domain is orange, Rhs domain is grey and the toxin domain is blue. The second sequence is VgrG15 where the entire sequence is purple. These colour schemes are retained for Tde16 and VgrG16 where the Rhs protein (Tde16) is coloured into the three domains and VgrG16 is purple.

Supp Table 5. MS/MS proteomic data from secretome analysis including whole cell lysate and supernatant samples from *A. baumannii* AB307-0294.

	**Peptide intensity**			**Number of unique peptides**		
	Av WCL	Av S/N	Ratio S/N:WCL	Av WCL	Av S/N	Ratio S/N:WCL
Tse15_Clade	2.23×10^{9}	1.62×10^{10}	7.3	7	9	1.3
Tse15_Cage	1.84×10^{10}	6.22×10^{9}	0.34	39.5	7	0.09
Tse15_Toxin	2.27×10^{9}	9.87×10^{9}	4.34	2.5	3.5	1.4
Tse16_Clade	3.50×10^{9}	7.90×10^{9}	2.25	10	9	0.9
Tse16_Cage	7.08×10^{9}	5.07×10^{8}	0.07	26.5	1	0.04
Tse16_Toxin	1.94×10^{9}	1.47×10^{10}	7.57	6	9.5	1.6
Tae17_Full	5.24×10^{9}	2.15×10^{10}	4.10	18	18.5	1.0
Hcp_Full	1.01×10^{11}	6.50×10^{11}	6.46	10	23	2.3
RpoB_Full	3.15×10^{11}	1.44×10^{9}	0.005	77	2.5	0.032

Av = average; WCL = whole cell lysate; S/N = supernatant

Table S6. Bacterial strains and plasmids used in this study.

Bacterial Strains	Description	Source
Escherichia coli		
AL3339	DH10B with vector pWH1266. For use as a negative control in T6SS killing assay.	[23]
AL3343	DH10B with pAL1265 encoding Tdi16 and Tai17 immunity proteins. Susceptible to killing by Tse15. For use in T6SS killing assay.	[23]
BTH101	*F-, cya-99, araD139, galE15, galK16, rpsL1 (Str r), hsdR2, mcrA1, mcrB1*. For use in bacterial two-hybrid assays.	Euromedex
DH5α	*fhuA2 lac(del)U169 phoA glnV44 Φ80' lacZ(del)M15 gyrA96 recA1 relA1 endA1 thi-1 hsdR17*. For general cloning purposes.	Bethesda Research Laboratories
DH10B	*F- mcrA Δ(mrr-hsdRMS-mcrBC) Φ80dlacZΔM15 ΔlacX74 endA1 recA1 deoR Δ(ara,leu)7697 araD139 galU galK nupG rpsL λ-*. For use in T6SS killing assay.	[43]
Rosetta™ 2	*F^- ompT $hsdS_B(r_B^-\ m_B^-)$ gal dcm pRARE2 (Cam^R)*. For recombinant protein expression.	Novagen
A. baumannii		
AB307-0294	Clinical wild-type isolate.	[44]
AL2751	Δ*vgrG15* deletion mutant of AB307_0294.	[23]
AL4693	Δ*vgrG16* Δ*vgrG17* Δ*tse15* deletion mutant of AB307_0294.	This study
AL4020	Markerless *tssM* deletion mutant of AB307_0294.	This study
AL4731	Δ*tssM* Δ*tsi15* AB307_0294 double mutant. Kanamycin resistance cassette replaces *tsi15* on AL4020 genome (Δ*tssM* AB307_0294).	This study
AL4734	Tse15 CC::FRT mutant of AB307_0294. A 36 bp insert including an FRT site is inserted in-frame into *tse15*, replacing nucleotides 1003 to 1038. Encoded Tse15 protein has the clade cleavage motif sequence SISYSIGAERVQ (Tse15 amino acids 335-346) replaced with KKFLFSRKYRNF.	This study
AL4745	Tse15 TC::FRT mutant of AB307_0294. A 36 bp insert including an FRT site is inserted in-frame into *tse15*, replacing nucleotides replacing nucleotides 4150 to 4185. Encoded Tse15 protein has the toxin cleavage motif sequence SDPNQWIDPKGL (Tse15 amino acids 1384-1395) replaced with KKFLFSRKYRNF.	This study
**Plasmids**	**Description**	**Source**
pAL1234	$Tse15_{tox}$ cloned into arabinose-inducible expression plasmid pBAD30. Insert contains ribosome binding site, start codon and nts 4186-4773 of *tse15* (encoding $Tse15_{tox}$).	[23]
pAL1265	Constitutive expression plasmid encoding T6SS immunity proteins Tdi16 and Tai17.	[23]
pAL1415	T6SS gene *vgrG15* from strain AB307_0294 cloned into expression vector pBASE. Recombinant gene expression under control of P_{tac} promoter.	[23]
pAL1416	T6SS gene *vgrG16* from strain AB307_0294 cloned into expression vector pBASE. Recombinant gene expression under control of P_{tac} promoter.	[23]
pAL1456	pBASE encoding chimera $VgrG16\_15_{831-1064}$, under control of P_{tac} promoter.	This study
pAL1457	EcoRI subclone of pAL1415, encodes *vgrG15* nucleotides 1-1589 (EcoRI site).	This study

pAL1458	EcoRI subclone of pAL1416, encodes *vgrG16* nucleotides 1-1598 (EcoRI site).	This study
pAL1471	pBASE expression plasmid encoding open reading frame representing VgrG15 amino acids 1 to 912. Constructed by ligating EcoRI-digested pAL1457 to a PCR product generated with BAP8527 and BAP8606.	This study
pAL1473	pBASE expression plasmid encoding open reading frame representing VgrG15 amino acids 1 to 1010. Constructed by ligating EcoRI-digested pAL1457 to EcoRI-digested PCR product generated with primers BAP8527and BAP8607.	This study
pAL1474	pBASE expression plasmid encoding open reading frame representing VgrG15 amino acids 1 to 1045. Constructed by ligating EcoRI-digested pAL1457 to EcoRI-digested PCR product generated with primers BAP8527 and BAP8612.	This study
pAL1547	pUT18C with insert encoding VgrG15 amino acids 530-1064, constructed using PCR product generated with primers BAP8806 and BAP8803.	This study
pAL1551	pKT25 with insert encoding Tse15 amino acids 2-1389, constructed using PCR product generated with primers BAP8800 and BAP8801.	This study
pAL1559	pUT18C with insert encoding VgrG15 amino acids 913-1064, constructed using PCR product generated with primers BAP8858 and BAP8859.	This study
pAL1569	pKT25 with insert encoding Tse15 amino acids 2-520, constructed using PCR product generated with primers BAP8800 and BAP8861.	This study
pAL1618	pUT18C with insert encoding VgrG15 amino acids 971-1045, constructed using PCR product generated with primers BAP9020 and BAP8977.	This study
pAL1627	pKT25 with insert encoding Tse15 amino acids 2-863, constructed using PCR product generated with primers BAP8800 and BAP 8980.	This study
pAL1628	pUT18C with insert encoding VgrG15 amino acids 976-1024, constructed using PCR product generated with primers BAP9029 and BAP9030.	This study
pAL1636	pKT25 with insert encoding Tse15 amino acids 380-1389, constructed using PCR product generated with primers BAP8979 and BAP8801.	This study
pAL1638	pKT25 with insert encoding Tse15 amino acids 2-542, constructed using PCR product generated with primers BAP8800 and BAP9033.	This study
pAL1639	pUT18C with insert encoding VgrG15 amino acids 976-1014, constructed using PCR product generated with primers BAP9029 and BAP9095.	This study
pAL1640	pKT25 with insert encoding Tse15 amino acids 2-646, constructed using PCR product generated with primers BAP8800 and BAP9090.	This study
pAL1656	pKT25 with insert encoding Tse15 amino acids 2-570, constructed using PCR product generated with primers BAP8800 and BAP9118.	This study
pAL1662	pKT25 with insert encoding Tse15 amino acids 31-570, constructed using PCR product generated with primers BAP9149 and BAP9118.	This study
pAL1663	pKT25 with insert encoding Tse15 amino acids 79-570, constructed using PCR product generated with primers BAP9150 and BAP9118.	This study

pAL1707	pBASE encoding mutated *vgrG15* with multiple alanine substitutions (L999A, N1000A, L1003A, S1004A, Q1007A, M1008A, L1011A). Constructed using BAP9144 and BAP9199.	This study
pAL1931	pUT18C with insert encoding VgrG15 amino acids 1010-1045, constructed using PCR product generated with primers BAP9031 and BAP8977.	This study
pET28:*tse15*	pET28a+ expression wildtype Tse15 with in-frame N-terminal StrepTag-II and linker (MA-WSHPQFEK-SA-) as well an in-frame C-terminal hexa-histidine tag.	This study
pET28:$tse15_{NN}$	pET28:*tse15* expressing Tse15(D1369N, D1391N).	This study
pET28:$tse15_{E342A}$	pET28:*tse15* expressing Tse15(E342A).	This study
pET28:$tse15_{K334A, S335A}$	pET28:*tse15* expressing Tse15(K334, S335A).	This study
pKT25	Bacterial adenylate cyclase two-hybrid vector, Kan^R	[42]
pUT18C	Bacterial adenylate cyclase two-hybrid vector, Amp^R	[42]
pKT25-zip	Positive control plasmid, bacterial adenylate cyclase two-hybrid system, Kan^R	[42]
pUT18C-zip	Positive control plasmid, bacterial adenylate cyclase two-hybrid system, Amp^R	[42]

Supp Table 7. Primers used in this study.

Primer Name	Sequence (5' – 3')	Description
BAP1430	TTAGCGGATCCTACCTGACG	Forward primer pBAD30, binds upstream of multiple cloning site. For Sanger sequencing and PCR amplification of Tse15tox from pAL1235
BAP1431	TTTTATCAGACCGCTTCTGC	Reverse primer pBAD30, binds downstream of multiple cloning site. For Sanger sequencing and PCR amplification of Tse15tox from pAL1235
BAP7767	TGCCACCTGACGTCTAAGAA	pBASE vector primer, reverse. Anneals upstream of cloning site
BAP8412	CCATGGCAGGTCGTAAATCACTGCATAATTCG	pBASE specific primer, anneals downstream of cloning site
BAP8527	GCTGGAGAAGGTTATGGTG	Forward primer. Anneals to *vgrG15* nucleotides 1489-1507 and *vgr16* nucleotides 1498-1526, upstream of EcoRI site
BAP8528	TTTTTAGTATCAGGAAGC	Reverse primer. Anneals to *vgrG15* nucleotides 1655-1638 and *vgr16* nucleotides 1664-1647, downstream of EcoRI site
BAP8581	GAATCTTTCCGAAAACTGGTG	Anneals to nucleotides 2441-2461 in vgrG15
BAP8582	CAGCATTGACTTGGAATTTCC	Anneals to nucleotides 2471-2491 in vgrG16
BAP8606	AAAAGAATTCTAACTTGTATTATCCTTCTCAA	For construction of pAL1471, encoding C-terminal truncation of VgrG15 (1-912)
BAP8607	AAAAGAATTCTAACTTTTCATTTGATCGGCAG	For construction of pAL1473, encoding C-terminal truncation of VgrG15 (1-1010)
BAP8612	AAAAGAATTCTATTGTGGTCTTAAATAATTTTGCG	For construction of pAL1474, encoding C-terminal truncation of VgrG15 (1-1045)
BAP8727	GCCACCATTATTTGATGCGGTTGCATGTAGCGGCGG	For alanine mutagenesis of *tae15* toxin region at position Y1442A. Forward primer to introduce mutation
BAP8728	CCGCCGCTACATGCAACCGCATCAAATAATGGTGGC	For alanine mutagenesis of Tae15 CT region at position Y1442A. Reverse primer to introduce mutation
BAP7342	CGAATTCATGAGGCACAGCG	Anneals to nucleotides 1597-1616 in *vgrG16*
BAP7648	CCGGAATTGCCAGCTGGGGC	For deletion of *tsi15* in strain AL4020. Forward primer to amplify central SOE-PCR product encoding kanamycin resistant gene *aph(3')* from pCR-Blunt II-TOPO (Invitrogen)
BAP7649	TTCAGAAGAACTCGTCAAGAAGGC	For deletion of *tsi15* in strain AL4020. Reverse primer to amplify kanamycin resistant gene *aph(3')* from pCR-Blunt II-TOPO Invitrogen)
BAP7767	TGCCACCTGACGTCTAAGAA	pBASE vector primer located upstream of cloning site
BAP8102	CCTTATTCTATTTATAAAGACCCAGTATGG	For deletion of *tsi15* in strain AL4020. Forward primer for upstream SOE-PCR product
BAP8103	AGGGCGCCCCAGCTGGCAATTCCGGCTTTATTCCATG TTTGCATTACTTAATCTCTC	For deletion of *tsi15* in strain AL4020. Reverse primer for upstream SOE-PCR product
BAP8104	GCCTTCTTGACGAGTTCTTCTGAAGAATAGTTGATTG GGATAAATTATTATGATTGTG	For deletion of *tsi15* in strain AL4020. Forward primer for downstream SOE-PCR product
BAP8105	CATACCGATATTCCATTCTTTTTCAATTGAAG	For deletion of *tsi15* in strain AL4020. Forward primer for downstream SOE-PCR product

BAP8800	AAACTGCAGGGGCTAAAGAGAATACACAACAGAAAG	For 2-hybrid analysis. Forward primer for construction of pAL1551, pAL1569, pAL1627, pAL1638, pAL1640, pAL1656
BAP8801	AAACCCGGGCTACCATTGATTTGGATCACTTACATAAC	For 2-hybrid analysis. Reverse primer for construction of pAL1551, pAL1636.
BAP8803	AAACCCGGGCTACTTCTTTGACCGTACAAAAGTATC	For 2-hybrid analysis. Reverse primer for construction of pAL1547
BAP8806	AAACTGCAGGCGAATTCATGAGGCCCAGCGCCATC	For 2-hybrid analysis. Forward primer for construction of pAL1547
BAP8858	AAAACTGCAGGGCATGGCTACAAAGCAGTGATTTTAAAC	For 2-hybrid analysis. Forward primer for construction of pAL1559
BAP8859	AAAACCCGGGCTACTTTTTTGACCGTACAAAAGTATC	For 2-hybrid analysis. Reverse primer for construction of pAL1559
BAP8861	AAAACCCGGGCTATTCATAACGTGCTAATGGCTCAGC	For 2-hybrid analysis. Reverse primer for construction of pAL1569
BAP8977	AAAACCCGGGCTATGTGGTCTTAAATAATTTTGCG	For 2-hybrid analysis. Reverse primer for construction of pAL1618 and pAL1931
BAP8979	AAACTGCAGGGATGATGCCATTTTCCAATATGATTC	For 2-hybrid analysis. Forward primer for construction of pAL1636
BAP8980	AAAACCCGGGTTAAAACAAATATTCGGCTTGATTC	For 2-hybrid analysis. Reverse primer for construction of pAL1548, pAL1627
BAP9020	AAACTGCAGGACTTTAGATAATTTAGCTGGC	For 2-hybrid analysis. Forward primer for construction of pAL1618
BAP9029	AAACTGCAGGGCTGGCCAAACAGTAGCCCAG	For 2-hybrid analysis. Forward primer for construction of pAL1628, pAL1639
BAP9030	AAAACCCGGGTTACATTTCAAGAGATTGACTTGG	For 2-hybrid analysis. Reverse primer for construction of pAL1931
BAP9031	AGTCTTGGTCAAATGGCGACG	For 2-hybrid analysis. Forward primer for construction of pAL1931
BAP9033	AAAACCCGGGGTTAATATTCATAGGTTCTTGTATGG	For 2-hybrid analysis. Reverse primer for construction of pAL1638
BAP9089	AAACTGCAGGGAATGGAAAAATTAAAGAAACG	For 2-hybrid analysis. Forward primer for construction of pAL1548
BAP9090	AAAACCCGGGGTTACTCTCGCCCATCAAAATCGATTTG	For 2-hybrid analysis. Reverse primer for construction of pAL1640
BAP9095	AAAACCCGGGTTACATTTGACCAAGACTTTTCATTTG	For 2-hybrid analysis. Reverse primer for construction of pAL1639
BAP9118	AAAACCCGGGGTTAAGCTTTGGCTTCAGTTGATTC	For 2-hybrid analysis. Reverse primer for construction of pAL1656, pAL1662, pAL1663
BAP9144	TGTAAAACGACGGCCAGTGAATTCGAGCTCGTT	For construction of pAL1707
BAP9149	AAACTGCAGGGGCGGACTTGCAAATGATCGCAAG	For 2-hybrid analysis. Forward primer for construction of pAL1662
BAP9150	AAACTGCAGGGCTCTATGCTACAGATAAAAAAACTG	For 2-hybrid analysis. Forward primer for construction of pAL1663
BAP9199	AAAATGGCCAAACAGTAGCCCAGATTAAAAATCAGGTAACTAATA CTGCCCTTAATACTGTTTCAAAACAAGCTGCTCTCAATGCAGCTG CCGATGCAGCGAAAAGTGCTGGTCAAATGG	For construction of pAL1707. Contains nucleotide changes to introduce alanine substitution at VgrG15 residues 999,1000, 1003, 1004, 1007, 1008 and 1011
BAP9216	CGTAAGCCTAATACACATAACGATG	For deletion of *tssM* in AB307_0294. SOE-PCR forward primer for amplifying region upstream of target region.
BAP9217	CATTCTATTCTCGTTCTAAGTTCAAAG	For deletion of *tssM* in AB307_0294. SOE-PCR reverse primer for amplifying region upstream of target region.

BAP9242	CTTTGAACTTAGAACGAGAATAGAATGGAAGTTCCTATACTTTCTAGAGAATAGGAACTTCCCGGAATTGCCAGC	For deletion of *tssM* in AB307_0294. Forward primer to amplify kanamycin resistant gene aph (3') from pCR-Blunt II-TOPO (Invitrogen). Includes an FRT site (underlined).
BAP9243	GAATGTAGAGGTTAGTTTCCATGTCATGAAGTTCCTATTCTCTAGAAAGTATAGGAACTTCTTCAGAAGAACTCG	For deletion of *tssM* in AB307_0294. Reverse primer to amplify kanamycin resistant gene aph (3') from pCR-Blunt II-TOPO (Invitrogen). Includes an FRT site (underlined).
BAP9218	ATGACATGGAAACTAACCTCTACATTC	For deletion of *tssM* in AB307_0294. SOE-PCR forward primer for amplifying region downstream of target region.
BAP9219	GCCCAGCTTCAGTTAATACTGC	For deletion of *tssM* in AB307_0294. SOE-PCR reverse primer for amplifying region downstream of target region.
BAP10046	GGACTAAAAGCTGTTGCTGCACAAGGCAAGG	For replacement of the *tse15* clade cleavage region with an in-frame FRT site in strain AB307_0294. SOE-PCR forward primer for amplifying region upstream of target region.
BAP10047	TTTCCAGATTGGGAATCAGCACCTTCACATGG	For replacement of the *tse15* clade cleavage region with an in-frame FRT site in strain AB307_0294. SOE-PCR reverse primer for amplifying region upstream of target region.
BAP10048	ATGTGAAGGTGCTGATTCCCAATCTGGAAAAAAGAAGTTCCTATTCTCTAGAAAGTATAGGAACTTCCCGGAATTGCCAGCTGGGG	For replacement of the *tse15* clade cleavage region with an in-frame FRT site in strain AB307_0294. Forward primer to amplify kanamycin resistant gene *aph(3')* from pCR-Blunt II-TOPO (Invitrogen). Includes an FRT site (underlined).
BAP10049	CCTATTTTCGGTAAATAGAAGTCTGCATGGAAGTTCCTATACTTTCTAGAGAATAGGAACTTCTTCAGAAGAACTCGTCAAGAAGGC	For replacement of the *tse15* clade cleavage region with an in-frame FRT site in strain AB307_0294. Reverse primer to amplify kanamycin resistant gene *aph(3')* from pCR-Blunt II-TOPO (Invitrogen). Includes an FRT site (underlined).
BAP10050	CATGCAGACTTCTATTTACCGAAAATAGG	For replacement of the *tse15* clade cleavage region with an in-frame FRT site in strain AB307_0294. SOE-PCR forward primer for amplifying region downstream of target region.
BAP10051	ATTTAAACCAATTGCATAAATATGCCCTGAACC	For replacement of the *tse15* clade cleavage region with an in-frame FRT site in strain AB307_0294. SOE-PCR reverse primer for amplifying region downstream of target region.
BAP9851	TGCAGATGGACAAATTGCTTCGAAAAG	For replacement of the *tse15* toxin cleavage region with an in-frame FRT site in strain AB307_0294. SOE-PCR forward primer for amplifying region upstream of target region.
BAP10063	TACATAACTAGAGGTATTCATACCACCCTC	For replacement of the *tse15* toxin cleavage region with an in-frame FRT site in strain AB307_0294. SOE-PCR reverse primer for amplifying region upstream of target region.
BAP10062	AGAGGGTGGTATGAATACCTCTAGTTATGTAAAGAAGTTCCTATTCTCTAGAAAGTATAGGAACTTCCCGGAATTGCCAGCTGGGG	For replacement of the *tse15* toxin cleavage region with an in-frame FRT site in strain AB307_0294. Forward primer to amplify kanamycin resistant gene *aph(3')* from pCR-Blunt II-TOPO (Invitrogen). Includes an FRT site (underlined).

BAP10064	CCAAACATTTCACCATAATTAAAACTATTGAAGTTCCTATACTTTCTAGAGAATAGGAACTTCTTCAGAAGAACTCGTCAAGAAGGC	For replacement of the *tse15* toxin cleavage region with an in-frame FRT site in strain AB307_0294. Reverse primer to amplify kanamycin resistant gene *aph(3')* from pCR-Blunt II-TOPO (Invitrogen). Includes an FRT site (underlined).
BAP10065	AATAGTTTTAATTATGGTGAAATGTTTGGTATTCC	For replacement of the *tse15* toxin cleavage region with an in-frame FRT site in strain AB307_0294. SOE-PCR forward primer for amplifying region downstream of target region.
BAP9178	TCTAATTAAAACTTAAAACCAGATTATCAAATT	For replacement of the *tse15* toxin cleavage region with an in-frame FRT site in strain AB307_0294. SOE-PCR reverse primer for amplifying region downstream of target region.
pKT25_fwd	GTCAAGGTGATCGGCAATG	Forward primer upstream of multiple cloning site in pKT25
pKT25_rev	GTTTTCCCAGTCACGACGTT	Reverse primer downstream of multiple cloning site in pKT25
pUT18C_fwd	ATGCTTCCGGCTCGTATG	Forward primer upstream of multiple cloning site in pUT18C
pUT18C_rev	TGTCTGTAAGCGGATGCC	Reverse primer upstream of multiple cloning site in pUT18C
UP	GTAAAACGACGGCCAGT	Universal primer. Used as reverse primer in pBASE, anneals downstream of MCS cloning site
